# Revisiting the genus *Hyphodermella* (Basidiomycota, Polyporales) with descriptions of three new species

**DOI:** 10.3897/mycokeys.128.172807

**Published:** 2026-02-16

**Authors:** Margarita Dueñas, María P. Martín, Sandra Nogal-Prata, M.Teresa Telleria

**Affiliations:** 1 Real Jardín Botánico, CSIC, Plaza de Murillo, 2, 28014 Madrid, Spain Universidad Villanueva Madrid Spain; 2 Universidad Villanueva, 28034 Madrid, Spain Real Jardín Botánico, CSIC Madrid Spain

**Keywords:** Corticioid fungi, morphological analysis, multigene phylogeny, Phanerochaetaceae, species complex, taxonomy

## Abstract

In this study, we revisited *Hyphodermella* with the aim of circumscribing the genus, exploring its species diversity, re-evaluating the usefulness of the morphological characters used for its identification, and providing a more precise interpretation of its geographical distribution. Specimens were studied using morphological characters and molecular phylogenetic analyses of the ITS, LSU, *RPB1*, and *RPB2* regions. The results of the concatenated phylogenetic trees based on two and four markers support the monophyly of *Hyphodermella* and the original description of the genus. Morphological and molecular evidence delimit six clades and one singleton within the *Hyphodermella* core; three of these correspond to species already described, *Hyphodermella corrugata*, *H. maunakeaensis*, and *H. rosae*, and three correspond to new species, *Hyphodermella paulusiae***sp. nov**., *H. ryvardenii***sp. nov**., and *H. salcedoae***sp. nov**., which are described in this paper.

## Introduction

This study is a continuation of the previous one carried out by Telleria et al. (2010) on *Hyphodermella* species in the Western Mediterranean, in which, based on morphological and molecular data, the identities of *Hyphodermella corrugata* (Fr.) J. Erikss. & Ryvarden and *H. rosae* (Bres.) Nakasone were delimited. According to the results of that study, *Hyphodermella* was considered to be monophyletic, and the morphology of the species studied was in agreement with the original description of the genus: “having resupinate, effuse, and crustose basidiome; hymenophore irregularly odontioid, with small aculei apically provided with incrusted, projecting cystidioid hyphae, visible under the lens; hyphal system monomitic, hyphae non-fibulatae; no true cystidia; basidia clavate ab. 30 µm long and spores ellipsoid, smooth, thin-walled, non-amyloid, ab. 10 × 5 µm” (Eriksson and Ryvarden 1976).

The genus remained monotypic until Gilbertson et al. (2001) described one more species, *H. maunakeaensis* Gilb. & Hemmes, from Hawaii, with smaller basidia and spores than *H. corrugata*. Based on morphological characters, Duhem (2010) transferred *Epithele ochracea* Bres. to *Hyphodermella ochracea* (Bres.) Duhem, and Duhem and Buyck (2011) described *H. brunneocontexta* Duhem & Buyck from the Mayotte Islands, with odontioid hymenophore, brown context, very thick-walled, densely interwoven, brown, and sometimes encrusted hyphae, but the results of an unpublished molecular analysis (B. Buyck, pers. com. 15 Mar 2022) suggest that the ITS sequence does not cluster this species with *H. corrugata* and *H. rosae*.

The integration of molecular data into taxonomic research and the exploration of new geographical areas led to a significant advance in the discovery of new species and the understanding of biodiversity. Based on ITS sequence analysis, more species were included in the genus: *Hyphodermella poroides* Y.C. Dai & C.L. Zhao, with poroid hymenophore (Zhao et al. 2017); *H. aurantiaca* C.L. Zhao (Wang and Zhao 2020); *H. pallidostraminea* Bukharova & Volobuev (Crous et al. 2021); and *H. zixishanensis* C.L. Zhao (Wang et al. 2021), with smooth hymenophore, without incrusted cystidioid hyphae, and with small basidia and spores. More recently, four new species: *H. laevigata* Yue Li & S.H. He and *H. tropica* Yue Li & S.H. He (Li et al. 2023), *H. suiae* Shan Shen, S.L. Liu & L.W. Zhou (Shen et al. 2023), *H. sinensis* Lu Wang & C.L. Zhao (Wang et al. 2024), and one new combination, *H. pallidovirens* (Bourdot & Galzin) K.H. Larsson & Spirin (Larsson et al. 2025) were also included in the genus.

Chen et al. (2021), in a broad multigene study of five markers — ITS, LSU, *RPB1*, *RPB2*, and *tef*1-α — on the phlebioid clade of Phanerochaetaceae, included *H. corrugata*, *H. rosae*, and *H. poroides*, considering *Hyphodermella* paraphyletic because *H. poroides* did not cluster in the core group. Shen et al. (2023) used *Hyphodermella* as an example of studies based on few species, in which taxa were placed in incorrect genera. They carried out a multigene phylogenetic study using the same markers as Chen et al. (2021) and erected the genus *Pseudohyphodermella* to accommodate *H. poroides*; they also transferred *H. aurantiaca* and *H. zixishanensis* to *Roseograndinia* and described *H. suiae* Shan Shen, S.L. Liu & L.W. Zhou, with smooth hymenophore and without encrusted cystidioid hyphae, because it clustered near, although not within, the core of *Hyphodermella*.

Other species described in *Hyphodermella*, *H. laevigata* Yue Li & S.H. He, with smooth hymenophore and without incrusted cystidioid hyphae; *H. tropica* Yue Li & S.H. He, with grandinioid hymenophore and lamprocystidia (Li et al. 2023); and *H. sinensis* Lu Wang & C.L. Zhao (Wang et al. 2024), with clamp-connections, do not group within the core *Hyphodermella*, so they would need to be revised to place them in the appropriate taxonomic position.

*Hyphodermella corrugata* was, for a long time, the only species considered in the genus. It has been described with some morphological variability, both in European specimens (Telleria et al. 2010) and in specimens from Argentina (Hjortstam and Ryvarden 1986) and Australia (Hjortstam et al. 2009), which were reported as *Hyphodermella aff. corrugata* due to the smaller size of their basidia and spores.

*Hyphodermella* species are associated with white rot on deciduous wood. After publishing the *H. rosae*ITS sequence (Telleria et al. 2010), its anamorph was identified as a pathogen on Rosaceae fruits (Babaeizad et al. 2012; Sayari et al. 2012) and *Philodendron* (Dell’Ollmo et al. 2024); it has also been isolated from ancient documents made of parchment (Paiva de Carvalho et al. 2016). Since the publication of ITS sequence, knowledge of the distribution of *H. rosae* has expanded to North America (Brazee et al. 2014; Floudas and Hibbett 2015; Dufresne et al. 2017) and Asia (Babaeizad et al. 2012; Ghobad-Nejhad and Hallenberg 2012; Sayari et al. 2012; Rahimlou et al. 2015; Aghayeva et al. 2018; Chen et al. 2021).

This study aims to circumscribe the genus, explore species diversity within *Hyphodermella*, re-evaluate the usefulness of the morphological characters used for its identification, and provide a more precise interpretation of its distribution. Specimens from other geographical areas, especially from the Southern Hemisphere, were included in this study; in addition to ITS and LSU, two additional molecular markers, *RPB1* and *RPB2*, were analyzed. Morphological characters used in species delimitation were also analyzed.

## Materials and methods

### Taxon sampling and morphological study

In addition to the 21 specimens included in Telleria et al. (2010), we expanded this study with specimens extensively collected in the Valdivian Rainforest of Chile. The initials JFL correspond to J. Fernández-López, MD to M. Dueñas, MPM to María P. Martín, and Tell. to M.T. Telleria. We also included collections from Australia, Hawaii, Nepal, South America, New Zealand, and the Canary Islands identified as *H. corrugata*, deposited in the CANB, MA-Fungi, O, and PDD herbaria, as well as the lectotype of *H. ochracea* (S) and one paratype of *H. maunakeaensis* (CFMR). Herbarium acronyms follow Index Herbariorum (http://sweetgum.nybg.org/science/ih/). Permission to extract DNA was granted. *H. brunneocontexta*, *H. laevigata*, *H. tropica*, and *H. sinensis* were not included in this study because their morphological characters and previous molecular analyses do not place them within the *Hyphodermella* core.

For morphological analysis, a total of 49 specimens were used (Table 1). Colors of dried basidiomata were given according to the ISCC–NBS Centroid Color Charts (Kelly and Judd 1976). Microscopic observations were performed using sections prepared with the aid of a Nikon SMZ 1000 stereomicroscope, mounted in 3% KOH and 1% aqueous Congo Red solutions, and examined at magnifications of up to 1250× using an Olympus BX51 microscope. Line drawings were made with a Leica DM2500 light microscope equipped with a drawing tube. The length and width of 10 spores and 10 basidia, as well as spore morphology, were analyzed. Mean values for the length (L) and width (W) of basidia and spores, as well as the Q value (length/width ratio), were calculated for each specimen. Additional morphological measurements were performed to provide a general description of the new species.

**Table 1.** Species and specimens included in the morphological study, with their herbarium or collector number and locality, size, and mean values of basidia and spores, and the Q (length/width) ratio of spores.

Species/specimens	Country	Basidia	Spores	Mean basidia	Mean spores	Q
***Hyphodermella corrugata*** (Fr.) J. Erikss. & Ryvarden		**30–61(–70) × 5–11**	**7–11 × 5–8**	**42.4 × 7.4**	**8.9 × 6.0**	**1.5**
F-89175 [*H. ochracea* (Bres.) Duhem, **lectotype**]	Italy	30–41 × 7–10	7–10 × 5–7	31.4 × 8.3	8.7 × 5.5	1.6
MA-Fungi 5527, 817Tell.	Morocco	35–55 × 8–11	8–10 × 5–7	45.2 × 8.8	8.9 × 6.2	1.4
MA-Fungi 5529, 759Tell.	Morocco	35 × 6	8–11 × 5–6	35 × 6	8.5 × 5.5	1.5
MA-Fungi 7653, 4638Tell.	Spain	45–54 × 9–11	7–10 × 6–7	50 × 6	9.3 × 6.5	1.4
MA-Fungi 11076, 3349MD	Spain	35–45 × 6–7	8.5–10.5 × 5–6	41.5 × 9.7	9.3 × 5.8	1.6
MA-Fungi 24238, 3969IM	Portugal	(35–)40–42 × 6–7	8–10 × 5–6	39.8 × 6.4	8.7 × 5.3	1.6
MA-Fungi 26185, 11457Tell.	Portugal	44–50 × 6–8	9–11 × 6–7	48.7 × 7.2	10.2 × 6.5	1.6
MA-Fungi 26186, 11459Tell.	Portugal	(38–)47–60 × 7–8	8.5–11 × 6–7	51.4 × 7.2	9.2 × 6.5	1.4
MA-Fungi 38073, 3500IS	Spain	45–51(–54) × 7–8	9–11 × 5–7.5	49 × 7.6	9.4 × 6.3	1.5
MA-Fungi 39201, 73Tell.	Spain	45–47 × 10–11	7–10 × 6–7	46 × 10.5	8.2 × 6.5	1.3
MA-Fungi 61382, 13285Tell.	France	37–47 × 6–7	8.5–11 × 6.5–7	37.2 × 6.8	9.3 × 6.7	1.4
MA-Fungi 61395, 13299Tell.	France	35–47 × 6–8	8–9 × 5–6.5	39.4 × 6.8	8.4 × 6	1.4
MA-Fungi 94244, 14453MD	Chile	40–52 × 5–6	8–9 × 5–6.5	45.5 × 5.7	8.6 × 5.7	1.5
MA-Fungi 94247, 14462MD	Chile	35–43 × 6–7	8–9 × 5–6	38.7 × 6.4	8.7 × 5.4	1.6
MA-Fungi 94256, 15126MD	Chile	30–47 × 5–10	7–10 × 5–6	34.3 × 7.2	7.8 × 5.8	1.3
MA-Fungi 94257, 15146MD	Chile	33–45(–70) × 6–8	7–9 × 5–6.5	43.2 × 7.1	8.5 × 5.8	1.5
MA-Fungi 94266, 15223MD	Chile	36–49 × 7–11	7.5–9 × 5–6.5	45.9 × 8.9	8.9 × 6.0	1.5
MA-Fungi 94271, 15247MD	Chile	35–53(–61) × 7–8	8–11 × 5–7	42.9 × 7.6	9.4 × 6.3	1.5
MA-Fungi 94291, 20535Tell.	Chile	32–52 × 7–9	8.5–10.5 × 5.5–7	37.9 × 7.8	9.2 × 6.2	1.5
MA-Fungi 94293, 20589Tell.	Chile	35–45 × 7–9	8–10 × 5–7	38.4 × 7.8	9.1 × 6.3	1.4
MA-Fungi 94296, 20593Tell.	Chile	38–55 × 6–8	8–11 × 6–7	45 × 6.9	9.7 × 6.4	1.5
MA-Fungi 94302, 20603Tell.	Chile	34–45 × 6–9	9–11 × 6–7	40.9 × 7.4	8.1 × 5.8	1.4
MA-Fungi 94272, 576JFL	Chile	39–53 × 6–9	8–8.5 × 5–6	45.4 × 7.7	8.7 × 6.4	1.4
MA-Fungi 94273, 582JFL	Chile	35–50 × 6–8	8–10 × 5.5–8	43.2 × 7.1	10.3 × 5.9	1.7
***Hyphodermella maunakeaensis*** Gilb. & Hemmes		**26–37 × 6–7**	**6.5–9 × 4.5–5**	**31.2 × 6.8**	**7.8 × 4.7**	**1.7**
CFMR, DEH-1641, **paratype**	USA: Hawaii	26–37 × 6–7	6.5–9 × 4.5–5	31.2 × 6.8	7.8 × 4.7	1.7
***Hyphodermella paulusiae*** M. Dueñas, Telleria & Martín, **sp. nov**.		**25–42 × 5–8**	**6–10 × 4–6**	**31.5 × 6.3**	**7.7 × 4.9**	**1.6**
CANB 889543, J.A. Curnow 6146	Australia	25–42 × 6–8	8–10 × 5–5.5	33.8 × 6.4	8.5 × 5.1	1.7
CANB 752189, H. Lepp 5014	Australia	26–30 × 7–8	7–8 × 4–5	28.3 × 7.5	7.4 × 4.5	1.6
CANB 752263, H. Lepp 5088	Australia	25–35 × 6–7	7–9 × 4–5	31.4 × 6.2	7.8 × 4.8	1.6
PDD 92259, BCP 4176, **holotype**	New Zealand	30–37 × 5–8	7–9 × 4–6	32.8 × 6.2	7.8 × 5	1.6
PDD 94113, BCP 3073	New Zealand	25–31 × 6	6–9 × 4.5–5	28 × 6	7.3 × 5	1.5
PDD 94114, BCP 3080	New Zealand	28–38 × 5–8	6–8.5 × 4–5.5	31.4 × 6.7	7.1 × 4.8	1.5
PDD 85550, PRJ 47	New Zealand	Not seen	6–10 × 4–5.5	–	7.6 × 4.8	1.6
***Hyphodermella rosae*** (Bres.) Nakasone		**21–42 × 5–9**	**5.5–9 × 3.5–6**	**28.1 × 6.8**	**7.2 × 4.7**	**1.5**
TR B/3785, **holotype**	Italy	28–42 × 7–9	7–8 × 5–6	31.1 × 7.3	7.6 × 5.7	1.3
MA-Fungi 1103	Spain	25–32 × 6–7	6.5–8 × 4–5	29.3 × 6.3	7 × 4.5	1.5
MA-Fungi 1556	Spain	21–26 × 7–8	7–8 × 4–5.5	24 × 7.3	7.3 × 4.7	1.5
MA-Fungi 14880, 2384MD	Spain	–	6.5–7 × 4–5	–	6.9 × 4.5	1.5
MA-Fungi 22291, 4302MD	Spain	30–34 × 5–7.5	7–9 × 4–5	32.2 × 6.5	7.7 × 4.7	1.6
MA-Fungi 22929, 4042MD	Spain	21–31 × 6–7	6–8 × 4–4.5	26.1 × 6.6	7.1 × 4.3	1.6
MA-Fungi 24292, 8196Tell.	Spain	21–38 × 6–8	7–8 × 4–5.5	27 × 6.8	7.7 × 4.8	1.6
MA-Fungi 36972, 10690Tell.	Spain	22–31 × 6–8	7–8 × 4–5.5	27 × 6.6	7.1 × 4.1	1.7
MA-Fungi 38071, 539IS	Spain	33–38 × 7–8	6.5–8 × 4.5–6	35 × 7.5	7 × 5.2	1.3
MA-Fungi 75541, 5703IM (*H. densa Melo & Hjortstam*) **paratype**	Portugal	18–28 × 5–7	5.5–7.5 × 3.5–4.5	21 × 6	6.4 × 4.7	1.4
***Hyphodermella ryvardenii*** M. Dueñas, Telleria & Martín, **sp. nov**.		**21–41(–46) × 5.5–9**	**7–9 × 4–6**	**28.7 × 6.6**	**7.5 × 4.8**	**1.6**
F-503386, Ryvarden 19509, **holotype**	Argentina	21–32 × 6–9	7–9 × 4–6	25.7 × 7.4	7.8 × 4.9	1.6
F-503387, Ryvarden 19825	Argentina	21 × 6	7 × 4.5	21 × 6	7 × 4.5	1.5
O-918413, Ryvarden 15568	Colombia	30–41(–46) × 6–8	7–8.5 × 4–4.5	36.6 × 6.7	7.8 × 4.4	1.8
O-918415, Ryvarden 15602	Colombia	28–35 × 5.5–7	7–8 × 4.5–5.5	31.6 × 6.3	7.4 × 5.2	1.4
***Hyphodermella salcedoae*** M. Dueñas, Telleria & Martín, **sp. nov**.		**30–52 × 5–8**	**8–10 × 5–7**	**44.7 × 6.6**	**9.4 × 6.0**	**1.6**
MA-Fungi 92643, 11452MD, **holotype**	Spain: Canary Islands	30–52 × 5–8	8.5–10 × 5–7	39.6 × 6.2	9.2 × 6	1.5
MA-Fungi 92646, 17082Tell.	Spain: Canary Islands	44–52 × 5–8	8–10 × 5–6.5	47.5 × 6.8	9.5 × 6	1.6
***Hyphodermella*** sp.						
O-903664, Ryvarden 18929	Nepal	29–43 × 5–7	6–8 × 4–5	35.4 × 6.1	7.1 × 4.6	1.5

### DNA extraction, PCR, and sequencing

Genomic DNA was isolated from 80 specimens, including the type specimen of *Hyphodermella ochracea* and one paratype of *H. maunakeaensis*, using the DNeasy Plant Mini Kit (Qiagen, Hilden, Germany), following the manufacturer’s instructions, except that samples were incubated in lysis buffer overnight at 55 °C and the elution buffer was preheated to 60 °C. For some recalcitrant specimens, FTA Indicating Micro Cards (Cat. No. WB120211, Whatman, Maidstone, England) were used for DNA isolation following the protocol of Telleria et al. (2014).

We use the term “marker” to denote any contiguous region of DNA (coding and non-coding). Four markers—the Internal Transcribed Spacer (ITS), which includes ITS1–5.8S–ITS2 nrDNA (fungal barcode; Schoch et al. 2012), the nuclear ribosomal large subunit (LSU nrDNA), the gene region for the second-largest subunit of RNA polymerase II (*RPB2*), and the gene region for the largest subunit of RNA polymerase II (*RPB1*)—were amplified with the primer pairs ITS5/ITS4 (White et al. 1990), LR0R/LR5 (Vilgalys and Hester 1990), bRPB2-6F/bRPB2-7.1R (Liu et al. 1999), and RPB1-Af/RPB1-Cr (Stiller and Hall 1997; Matheny et al. 2002), respectively. When the primer pair ITS5/ITS4 failed, ITS amplification was performed in two parts, using the primer pair ITS5/ITS2 (White et al. 1990) to obtain the ITS1 region, including the start of 5.8S, and the primer pair ITS3/ITS4 (White et al. 1990) to obtain the ITS2 region and the final part of 5.8S.

Amplification reactions were carried out in individual reactions to a final volume of 25 µl using Ready-To-Go® PCR Beads (Amersham Biosciences, Little Chalfont, Buckinghamshire, UK), as described by Winka et al. (1998), or the Biotools™ Mouse Direct PCR Kit (B4001; Selleckchem, Munich, Germany). Negative controls lacking fungal DNA were included to check for contamination of reagents.

Amplified DNA fragments were purified using the QIAquick Gel Extraction Kit (QIAGEN, Valencia, California, USA) or Illustra ExoProStar™ (GE Healthcare, Buckinghamshire, UK), following the manufacturers’ instructions. When more than 20 ng/µl of product was obtained, both strands were sequenced separately using the primers described above by Macrogen, Inc. (Madrid, Spain). Sequences were edited using Sequencer 5.1 software (Gene Codes Corporation, Ann Arbor, MI, USA) by overlapping the unidirectional reads. BLAST searches using the megablast option were performed to compare the obtained sequences against those in the EMBL/GenBank/DDBJ databases.

The newly generated sequences, together with those available in the EMBL/GenBank/DDBJ databases up to 2022, obtained mainly from our previous study (Telleria et al. 2010) and from studies by other authors, as well as some unpublished sequences (Table 2), were aligned separately using Se-Al v2.0a11 Carbon (Rambaut 2002). Five species of *Phanerochaete* and *Pirex concentricus* from Floudas and Hibbett (2015) were included as outgroup taxa.

**Table 2.** Species and specimens used to reconstruct the phylogenetic trees, with their herbarium or isolate numbers, locality, and GenBank accession numbers. GenBank accession numbers in boldface refer to sequences generated for this study.

Species/Specimen/Isolate	Country	GenBank Accesion number				References
		ITS	LSU	*RPB*2	*RPB*1	
***Hyphodermella corrugata*** (Fr.) J. Erikss. & Ryvarden						
Achao 41	Chile	KF638510	–	–	–	Ortiz et al. (2014)
F-89175, [*H. ochracea* (Bres.) Duhem, **lectotype**]	Italy	–	–	**MZ147895**	**MZ147936**	This study
MA-Fungi 5527, 817Tell.	Morocco	FN600372	JN939597	–	–	Telleria et al. (2010)
MA-Fungi 5529, 759Tell.	Morocco	FN600381	JN939583	**MZ147905**	–	Telleria et al. (2010); this study
MA-Fungi 7653, 4638Tell.	Spain	FN600375	JN939593	**MZ147910**	**MZ147957**	Telleria et al. (2010); this study
MA-Fungi 11076, 3349MD	Spain	FN600376	JN939590	–	–	Telleria et al. (2010)
MA-Fungi 24238, 3969IM	Portugal	FN600378	JN939586	**MZ147897**	**MZ147955**	Telleria et al. (2010); this study
MA-Fungi 26185, 11457Tell.	Portugal	FN600382	JN939582	**MZ147907**	–	Telleria et al. (2010); this study
MA-Fungi 26186, 11459Tell.	Portugal	FN600379	JN939585	**MZ147909**	–	Telleria et al. (2010); this study
MA-Fungi 38073, 3500IS	Spain	FN600377	JN939587	–	–	Telleria et al. (2010)
MA-Fungi 39201, 73Tell.	Spain	FN600374	JN939594	–	–	Telleria et al. (2010)
MA-Fungi 61382, 13285Tell.	France	FN600373	JN939596	**MZ147906**	**MZ147956**	Telleria et al. (2010); this study
MA-Fungi 61395, 13299Tell.	France	FN600380	JN939584	**MZ147908**	**MZ147953**	Telleria et al. (2010); this study
MA-Fungi 94244, 14453MD	Chile	**MZ147632**	**MZ147712**	–	–	This study
MA-Fungi 94245, 14454MD	Chile	**MZ147633**	**MZ147711**	–	–	This study
MA-Fungi 94246, 14458MD	Chile	**MZ147634**	**MZ147713**	–	–	This study
MA-Fungi 94247, 14462MD	Chile	**MZ147635**	**MZ147714**	–	–	This study
MA-Fungi 94248, 14646MD	Chile	**MZ147693**	**MZ147738**	–	–	This study
MA-Fungi 94249, 15105MD	Chile	**MZ147636**	**MZ147715**	–	–	This study
MA-Fungi 94250, 15106MD	Chile	**MZ147637**	**MZ147716**	–	–	This study
MA-Fungi 94251, 15118MD	Chile	**MZ147638**	**MZ147717**	–	–	This study
MA-Fungi 94252, 15121MD	Chile	**MZ147639**	**MZ147718**	**MZ147901**	–	This study
MA-Fungi 94253, 15123MD	Chile	**MZ147640**	**MZ147719**	–	–	This study
MA-Fungi 94254, 15124MD	Chile	**MZ147641**	**MZ147720**	–	–	This study
MA-Fungi 94255, 15125MD	Chile	**MZ147642**	**MZ147721**	–	–	This study
MA-Fungi 94256, 15126MD	Chile	**MZ147643**	**MZ147722**	**MZ147894**	**MZ147940**	This study
MA-Fungi 94257, 15146MD	Chile	**MZ147644**	**MZ147723**	**MZ147891**	**MZ147954**	This study
MA-Fungi 94258, 15147MD	Chile	**MZ147645**	**MZ147724**	–	–	This study
MA-Fungi 94259, 15148MD	Chile	**MZ147646**	**MZ147725**	–	–	This study
MA-Fungi 94260, 15149MD	Chile	**MZ147647**	**MZ147726**	–	–	This study
MA-Fungi 94261, 15186MD	Chile	**MZ147648**	**MZ147727**	–	**MZ147948**	This study
MA-Fungi 94262, 15194MD	Chile	**MZ147649**	**MZ147728**	–	**MZ147937**	This study
MA-Fungi 94263, 15213MD	Chile	**MZ147650**	**MZ147729**	–	–	This study
MA-Fungi 94264, 15217MD	Chile	**MZ14765**	**MZ147730**	–	–	This study
MA-Fungi 94265, 15222MD	Chile	**MZ147652**	**MZ147731**	–	–	This study
MA-Fungi 94266, 15223MD	Chile	**MZ147653**	**MZ147732**	**MZ147904**	**MZ147938**	This study
MA-Fungi 94267, 15232MD	Chile	**MZ147654**	**MZ147733**	–	–	This study
MA-Fungi 94268, 15233MD	Chile	**MZ147655**	**MZ147734**	–	**MZ147950**	This study
MA-Fungi 94269, 15240MD	Chile	**MZ147656**	**MZ147735**	–	**MZ147942**	This study
MA-Fungi 94270, 15243MD	Chile	**MZ147657**	**MZ147736**	–	**MZ147949**	This study
MA-Fungi 94271, 15247MD	Chile	**MZ147658**	**MZ147737**	**MZ147892**	**MZ147944**	This study
MA-Fungi 94272, 576JFL	Chile	**MZ147694**	**MZ147767**	**MZ147900**	**MZ147946**	This study
MA-Fungi 94273, 582JFL	Chile	**MZ147695**	**MZ147768**	**MZ147899**	**MZ147945**	This study
MA-Fungi 94274, 3484MPM	Chile	**MZ147689**	**MZ147739**	–	–	This study
MA-Fungi 94275, 3492MPM	Chile	**MZ147690**	–	–	–	This study
MA-Fungi 94276, 3498MPM	Chile	**MZ147691**	–	–	–	This study
MA-Fungi 94277, 3511MPM	Chile	**MZ147692**	**MZ147758**	**MZ147911**	–	This study
MA-Fungi 94278, 19693Tell.	Chile	**MZ147659**	–	–	–	This study
MA-Fungi 94279, 19696Tell.	Chile	**MZ147660**	**MZ147740**	–	–	This study
MA-Fungi 94280, 19704Tell.	Chile	**MZ147661**	–	–	–	This study
MA-Fungi 94281, 19949Tell.	Chile	**MZ147662**	–	–	–	This study
MA-Fungi 94307, 19951Tell.	Chile	**MZ147663**	**MZ147741**	–	–	This study
MA-Fungi 94282, 19957Tell.	Chile	**MZ147664**	**MZ147742**	–	–	This study
MA-Fungi 94283, 19963Tell.	Chile	**MZ147665**	**MZ147743**	–	–	This study
MA-Fungi 94284, 19967Tell.	Chile	**MZ147666**	–	–	–	This study
MA-Fungi 94285, 20022Tell.	Chile	**MZ147667**	**MZ147744**	–	–	This study
MA-Fungi 94286, 20023Tell.	Chile	**MZ147668**	**MZ147745**	–	–	This study
MA-Fungi 94287, 20041Tell.	Chile	**MZ147669**	**MZ147746**	–	–	This study
MA-Fungi 94288, 20472Tell.	Chile	**MZ147688**	**MZ147747**	–	–	This study
MA-Fungi 94289, 20525Tell.	Chile	**MZ147670**	**MZ147748**	–	–	This study
MA-Fungi 94290, 20530Tell.	Chile	**MZ147671**	**MZ147749**	–	–	This study
MA-Fungi 94291, 20535Tell.	Chile	**MZ147672**	**MZ147750**	**MZ147898**	**MZ147941**	This study
MA-Fungi 94292, 20588Tell.	Chile	**MZ147673**	**MZ147751**	–	**MZ147952**	This study
MA-Fungi 94293, 20589Tell.	Chile	**MZ147674**	**MZ147752**	**MZ147896**	**MZ147951**	This study
MA-Fungi 94294, 20591Tell.	Chile	**MZ147675**	**MZ147753**	–	–	This study
MA-Fungi 94295, 20592Tell.	Chile	**MZ147676**	**MZ147754**	–	–	This study
MA-Fungi 94296, 20593Tell.	Chile	**MZ147677**	**MZ147755**	**MZ147903**	**MZ147943**	This study
MA-Fungi 94297, 20595Tell.	Chile	**MZ147678**	**MZ147756**	**MZ147893**	**MZ147939**	This study
MA-Fungi 94298, 20597Tell.	Chile	**MZ147679**	**MZ147757**	–	–	This study
MA-Fungi 94299, 20598Tell.	Chile	**MZ147680**	**MZ147759**	–	–	This study
MA-Fungi 94300, 20599Tell.	Chile	**MZ147681**	**MZ147760**	–	–	This study
MA-Fungi 94301, 20600Tell.	Chile	**MZ147682**	**MZ147761**	–	–	This study
MA-Fungi 94302, 20603Tell.	Chile	**MZ147683**	**MZ147762**	**MZ147902**	**MZ147947**	This study
MA-Fungi 94303, 20604Tell.	Chile	**MZ147684**	**MZ147763**	–	–	This study
MA-Fungi 94304, 20605Tell.	Chile	**MZ147685**	**MZ147764**	–	–	This study
MA-Fungi 94305, 20607Tell.	Chile	**MZ147686**	**MZ147765**	–	–	This study
MA-Fungi 94306, 20608Tell.	Chile	**MZ147687**	**MZ147766**	–	–	This study
***Hyphodermella maunakeaensis*** Gilb. & Hemmes						
CFMR, DEH-1641, **paratype**	USA: Hawaii	**MZ147698**	**MZ147769**	**MZ147890**	**MZ147972**	This study
***Hyphodermella paulusiae*** M. Dueñas, Telleria & Martín, **sp. nov**.						
CANB 889543, J.A. Curnow 6146	Australia	**MZ147706**	**MZ147777**	**MZ147919**	**MZ147968**	This study
CANB 752189, H. Lepp 5014	Australia	**MZ147704**	**MZ147775**	**MZ147921**	**MZ147969**	This study
CANB 752263, H. Lepp 5088	Australia	**MZ147705**	**MZ147776**	**MZ147920**	**MZ147970**	This study
PDD85550, PRJ 47	New Zealand	**MZ147702**	**MZ147781**	**MZ147917**	–	This study
PDD92259, BCP 4176, **holotype**	New Zealand	**MZ147703**	**MZ147778**	**MZ147915**	–	This study
PDD94113, BCP 3073	New Zealand	**MZ147701**	**MZ147779**	**MZ147916**	–	This study
PDD94114, BCP 3080	New Zealand	**MZ147700**	**MZ147780**	**MZ147918**	**MZ147971**	This study
***Hyphodermella rosae*** (Bres.) Nakasone						
FP 150552	USA: Hawaii	KP134978	KP135223	KP150552	–	Floudas and Hibbett (2015)
KUC11033	South Korea	KJ714014	–	–	–	Jang et al. (2015)
MA-Fungi 1103	Spain	FN600384	–	MZ147929	–	Telleria et al. (2010)
MA-Fungi 1556	Spain	FN600385	JN939592	MZ147928	–	Telleria et al. (2010)
MA-Fungi 14880, 2384MD	Spain	FN600387	–	MZ147933	–	Telleria et al. (2010)
MA-Fungi 22291, 4302MD	Spain	FN600386	JN939591	MZ147925	–	Telleria et al. (2010)
MA-Fungi 22929, 4042MD	Spain	FN600391	JN939580	MZ147932	MZ147964	Telleria et al. (2010)
MA-Fungi 24292, 8196Tell.	Spain	FN600388	JN940190	MZ147930	MZ147963	Telleria et al. (2010)
MA-Fungi 36972, 10690Tell.	Spain	FN600390	JN939581	MZ147931	MZ147962	Telleria et al. (2010)
MA-Fungi 38071, 5391IS	Spain	FN600389	JN939588	MZ147926	MZ147966	Telleria et al. (2010)
MA-Fungi 75541, 5703IM (*Hyphodermella densa*, **paratype**)	Portugal	**FN600383**	**JN939595**	**MZ147927**	**MZ147965**	Telleria et al. (2010)
TR B/3785, **holotype**	Italy	**FN600392**	–	–	–	Telleria et al. (2010)
Strain V2ER12	France	KT692555	–	–	–	Comby et al. (2016)
Isolate AB1	Iran	JN593086	–	–	–	Sayari et al. (2012)
CFMR, DLL2011-177	USA	KJ140672	–	–	–	Brazee et al. (2014)
CFMR, DLL2011-243	USA	KJ140730	–	–	–	Brazee et al. (2014)
Isolate H16	Iran	–	KM103081	–	–	Rahimlou et al. (2015)
Isolate LSPQ-NSM-019	Canada	KU761148	KU761027	–	–	Dufresne et al. (2017)
Isolate P1 (from fruit)	Iran	–	KM103082	–	–	Rahimlou et al. (2015)
Isolate 15AZE093	Azerbaijan	MH062974	–	–	–	Aghayeva et al. (2018)
Isolate 15AZE114	Azerbaijan	MH062977	–	–	–	Aghayeva et al. (2018)
strain A84	Portugal	KT898650	–	–	–	Paiva de Carvalho et al. (2016)
***Hyphodermella ryvardenii*** M. Dueñas, Telleria & Martín, **sp. nov**.						
F-503386, Ryvarden 19509, **holotype**	Argentina	**MZ147710**	**MZ147772**	**MZ147912**	**MZ147958**	This study
O-F-503387, Ryvarden 19825	Argentina	**MZ147709**	**MZ147773**	**MZ147913**	**MZ147959**	This study
O-918413, Ryvarden 15568	Colombia	**MZ147707**	**MZ147774**	–	**MZ147920**	This study
O-918415, Ryvarden 15602	Colombia	**MZ147708**	–	**MZ147914**	**MZ147921**	This study
***Hyphodermella salcedoae*** M. Dueñas, Telleria & Martín, **sp. nov**.						
MA-Fungi 92643, 11452MD, **holotype**	Spain, Canary Islands	**MZ147697**	**MZ147770**	**MZ147923**	**MZ147935**	This study
MA-Fungi 92646, 17082Tell.	Spain, Canary Islands	MZ147696	MZ147771	MZ147922	MZ147934	This study
***Hyphodermella*** sp.						
O-903664, Ryvarden 18929	Nepal	MZ147699	MZ147782	MZ147924	MZ147967	This study
***Roseograndinia aurantiaca*** (C.L. Zhao) Yue Li & S.H. He						
CLZhao 10480	China	MW209022	MW209011	–	–	Zhao et al. (2017)
CLZhao 10487, **holotype**	China	MW209023	MW209012	–	–	Zhao et al. (2017)
CLZhao 10491	China	MW209024	MW209013	–	–	Zhao et al. (2017)
CLZhao 10500	China	MW209025	MW209014	–	–	Zhao et al. (2017)
CLZhao 10508	China	MW209026	MW209015	–	–	Zhao et al. (2017)
CLZhao 10510	China	MW209027	MW209016	–	–	Zhao et al. (2017)
CLZhao 10519	China	MW209028	MW209017	–	–	Zhao et al. (2017)
CLZhao 10521	China	MW209029	MW209018	–	–	Zhao et al. (2017)
CLZhao 10523	China	MW209030	MW209019	–	–	Zhao et al. (2017)
CLZhao 10525	China	MW209031	–	–	–	Zhao et al. (2017)
CLZhao 10530	China	MW209032	MW209020	–	–	Zhao et al. (2017)
CLZhao 10551	China	MW209033	MW209021	–	–	Zhao et al. (2017)
***Hyphodermella pallidostraminea*** Bukharova & Volobuev						
LE 286968, **holotype**	Russia	OK138912	*OK138912	–	–	Crous et al. (2021)
***Pseudohyhodermella poroides*** (Y.C. Dai & C.L. Zhao) Shan Shen, S.L. Liu & L.W. Zhou						
Dai 10848	China	KX008368	KX011853	–	–	Zhao et al. (2017)
Dai 12045, **holotype**	China	KX008367	KX011852	–	–	Zhao et al. (2017)
***Roseograndinia zixishanensis*** (C.L. Zhao) Yue Li & S.H. He						
CLZhao 7124	China	MZ305276	–	–	–	Wang et al. (2021)
CLZhao 7129	China	MZ305277	MZ305286	–	–	Wang et al. (2021)
CLZhao 7159	China	MZ305278	MZ305287	–	–	Wang et al. (2021)
CLZhao 7204	China	MZ305279	MZ305288	–	–	Wang et al. (2021)
CLZhao 7206	China	MZ305280	MZ305289	–	–	Wang et al. (2021)
CLZhao 7228	China	MZ305281	MZ305290	–	–	Wang et al. (2021)
CLZhao 7412	China	MZ305282	MZ305291	–	–	Wang et al. (2021)
CLZhao 7433	China	MZ305283	MZ305292	–	–	Wang et al. (2021)
CLZhao 7535	China	MZ305284	–	–	–	Wang et al. (2021)
CLZhao 7718, **holotype**	China	MZ305285	MZ305293	–	–	Wang et al. (2021)
Basidiomycota sp.						
Castro 19-4	Chile	KF638516	–	–	–	Ortiz et al. (2014)
Outgroup:						
*Phanerochaete australis* Jülich						
HHB-7105-Sp	USA	KP135081	KP135240	KP134957	KP134840	Floudas and Hibbett (2015)
*Phanerochaete chrysosporium* Burds.						
HHB-6251-Sp, **type**	USA	KP135094	KP135246	KP134954	KP134842	Floudas and Hibbett (2015)
*Phanerochaete laevis* (Fr.) J. Erikss. & Ryvarden						
HHB-15519-Sp	USA	KP135149	KP135149	KP134952	KP134836	Floudas and Hibbett (2015)
*Phanerochaete magnoliae* (Berk. & M.A. Curtis) Burds.						
HHB-9829-Sp	USA	KP135089	KP135237	KP134955	KP134838	Floudas and Hibbett (2015)
*Phanerochaete pseudomagnoliae* Koker, Burds. & B.J.H. Janse						
PP-25, **type**	South Africa	KP135091	KP135250	KP134956	KP134839	Floudas and Hibbett (2015)
*Pirex concentricus* (Cooke & Ellis) Hjortstam & Ryvarden						
OSC-41587	USA	KP134984	KP135275	KP134940	KP134843	Floudas and Hibbett (2015)

### Parsimony, maximum likelihood, and Bayesian analyses

The congruence of the four datasets (ITS, LSU, *RPB2*, and *RPB1*) was evaluated using a preliminary parsimony bootstrap analysis with a heuristic search in PAUP*v4.0a (Swofford 2002), with default settings for stopping the analysis. Branch lengths equal to zero were collapsed into polytomies, and nonparametric bootstrap support (Felsenstein 1985) for each clade was assessed using the fast-step option with 10,000 replicates. Gaps were treated as missing data (Suwannasai et al. 2013). Trees were rooted with the six species mentioned above from Floudas and Hibbett (2015). Conflicts among datasets were considered following Hillis and Bull (1993), and the datasets were concatenated to reconstruct the phylogenetic trees. Because many specimens did not yield all markers and some markers were not available in the EMBL/GenBank/DDBJ databases, three concatenated datasets were prepared to improve phylogenetic resolution: dataset 1 (ITS + LSU), dataset 2 (*RPB2* + *RPB1*), and dataset 3 (ITS + LSU + *RPB2* + *RPB1*), except for two specimens from Colombia that included three markers.

To evaluate whether species were recovered as monophyletic groups, the three concatenated datasets were analyzed using maximum parsimony (MP), maximum likelihood (ML), and Bayesian inference. All trees were rooted with the six species from Floudas and Hibbett (2015). For ML and Bayesian analyses, PAUP v4.0a selected the best-fit model for each partition: ITS1, 5.8S, and ITS2 under the HKY+I+G model, and LSU, *RPB2*, and *RPB1* under the GTR+I+G model. ML analyses were conducted with PAUP v4.0a. Bayesian analyses were conducted using MrBayes 3.2 (Ronquist et al. 2012). Two independent analyses starting from different random trees were run for two million generations with 12 parallel chains. Trees and model scores were sampled every 100 generations, and the first 1,000 trees were discarded as burn-in before calculating the 50% majority-rule consensus tree and posterior probabilities of nodes.

Phylogenetic trees were visualized and edited using FigTree version 1.4.4 (http://tree.bio.ed.ac.uk/software/figtree). A combination of bootstrap proportions and posterior probabilities was used to assess support for individual nodes (Lutzoni et al. 2004; Wilson et al. 2012).

### Statistical tests of morphological characters

Basidia and spore morphology were analyzed to assess morphological variation among species. One-way ANOVA tests were performed using IBM SPSS statistical software among the seven *Hyphodermella* lineages delimited by molecular phylogenetic analyses. A *p*-value < 0.05 was considered significant, and a larger F statistic indicated more pronounced species separation. Tukey HSD post hoc analyses of basidia and spores were performed to assess variation in basidium and spore length, width, and spore Q values; clades represented by only one specimen were excluded from this analysis.

## Results

### New sequences and datasets

**Figure 1.**
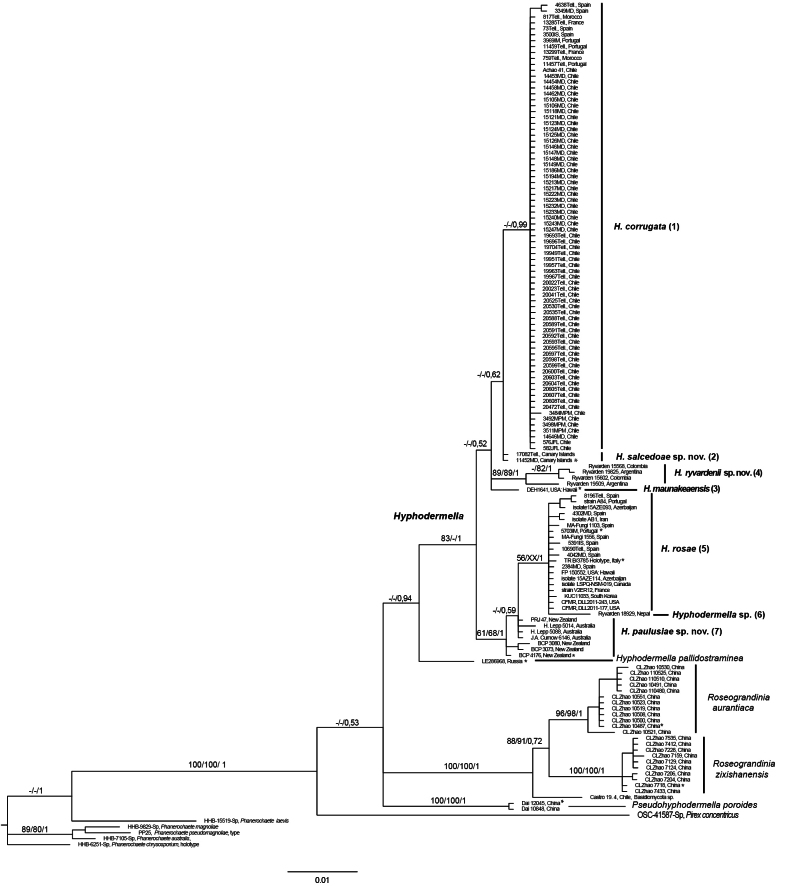
The 50% majority-rule consensus tree from a Bayesian analysis based on the concatenated ITS nrDNA dataset of *Hyphodermella* species. Five *Phanerochaete* species and *Pirex concentricus* were used as outgroup taxa. Maximum parsimony bootstrap percentages, maximum likelihood bootstrap percentages, and posterior probabilities are indicated at the branches. Type sequences are marked with *.

A total of 234 new sequences were generated in this study: 79 ITS, 72 LSU, 44 *RPB2*, and 39 *RPB1*. All sequences were deposited in GenBank under the accession numbers indicated in Table 2. Including outgroups, the final individual datasets contained 142 ITS sequences with 688 positions, 122 LSU sequences with 1,380 positions, 51 *RPB2* sequences with 702 positions, and 45 *RPB1* sequences with 1,318 positions. After preliminary parsimony bootstrapping using heuristic searches, the ITS (Fig. 1), LSU (data not shown), and *RPB2* (data not shown) trees showed similar terminal branches, whereas the *RPB1* tree differed in the placement of four specimens, two from Argentina (Ryvarden 19509, Ryvarden 19825) and two from Colombia (Ryvarden 15568, Ryvarden 15602), which grouped with the *Hyphodermella corrugata* clade in this marker (data not shown) instead of forming a unique clade as observed in the ITS, LSU, and *RPB2* analyses.

In the ITS tree (Fig. 1), the monophyly of the genus *Hyphodermella* was strongly supported by MP (83%) and PP (1.0), excluding *H. aurantiaca*, *H. pallidostraminea*, *H. poroides*, and *H. zixishanensis*, which did not belong to this genus.

**Figure 2.**
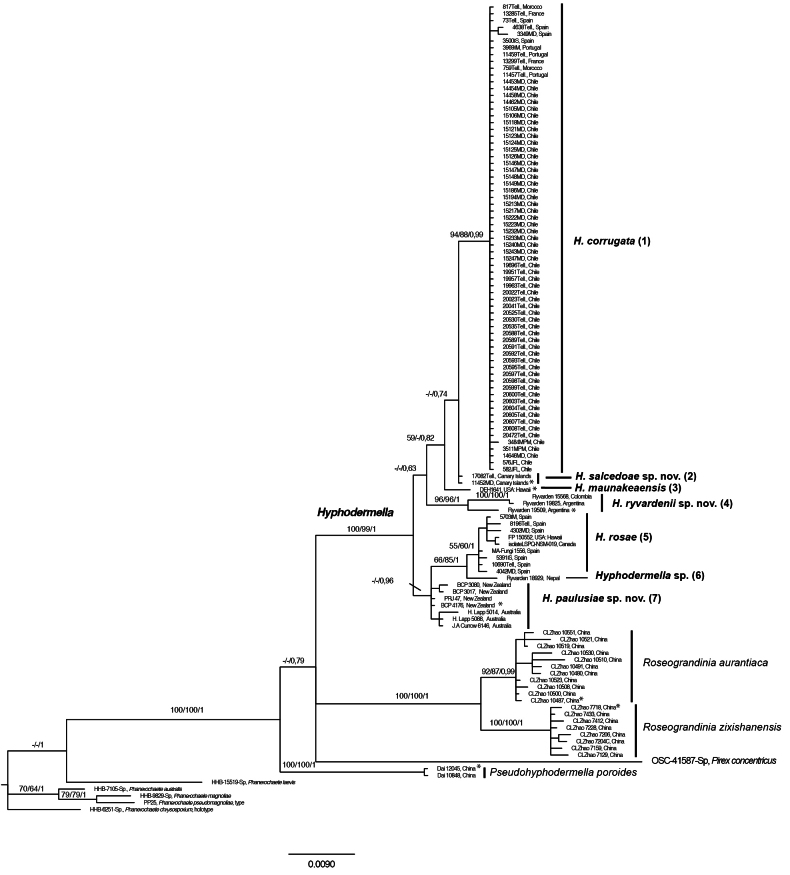
The 50% majority-rule consensus tree from a Bayesian analysis based on the concatenated ITS nrDNA and LSU nrDNA dataset of *Hyphodermella* species. Five *Phanerochaete* species and *Pirex concentricus* were used as outgroup taxa. Maximum parsimony bootstrap percentages, maximum likelihood bootstrap percentages, and posterior probabilities are indicated at the branches. Type sequences are marked with *.

**Figure 3.**
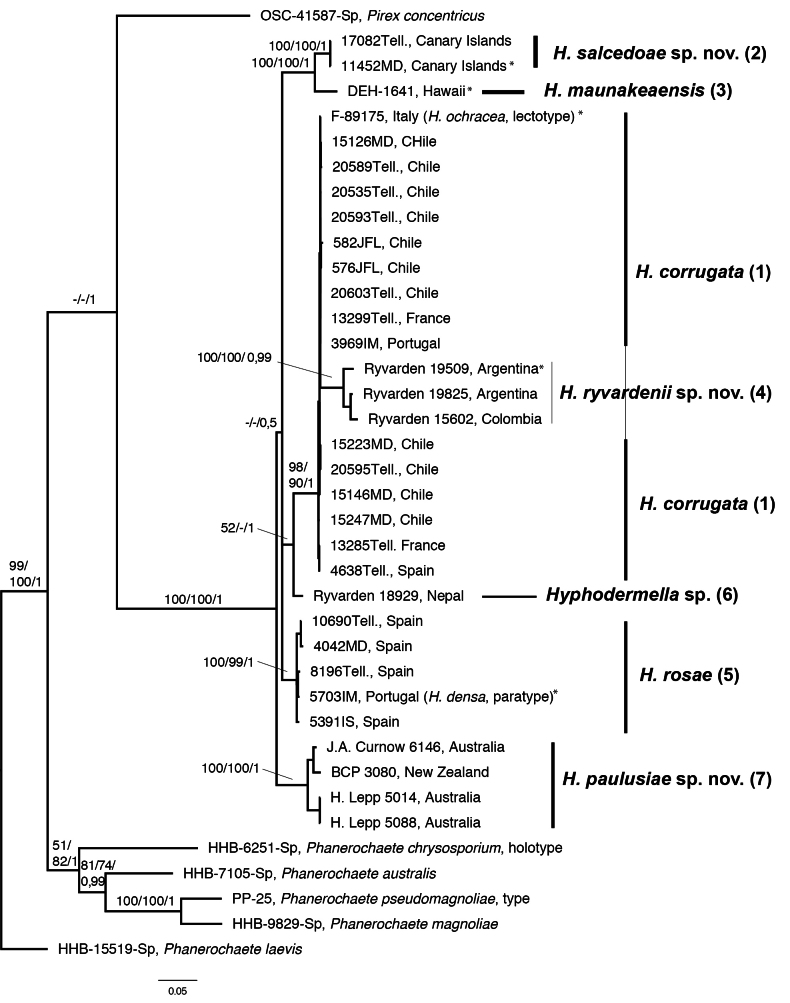
The 50% majority-rule consensus tree from a Bayesian analysis based on the concatenated dataset of *RPB2* and *RPB1* of *Hyphodermella* species. Five *Phanerochaete* species and *Pirex concentricus* were used as outgroups. Maximum parsimony bootstrap percentages, maximum likelihood bootstrap percentages, and posterior probabilities are indicated at the branches. Type sequences are marked with *.

**Figure 4.**
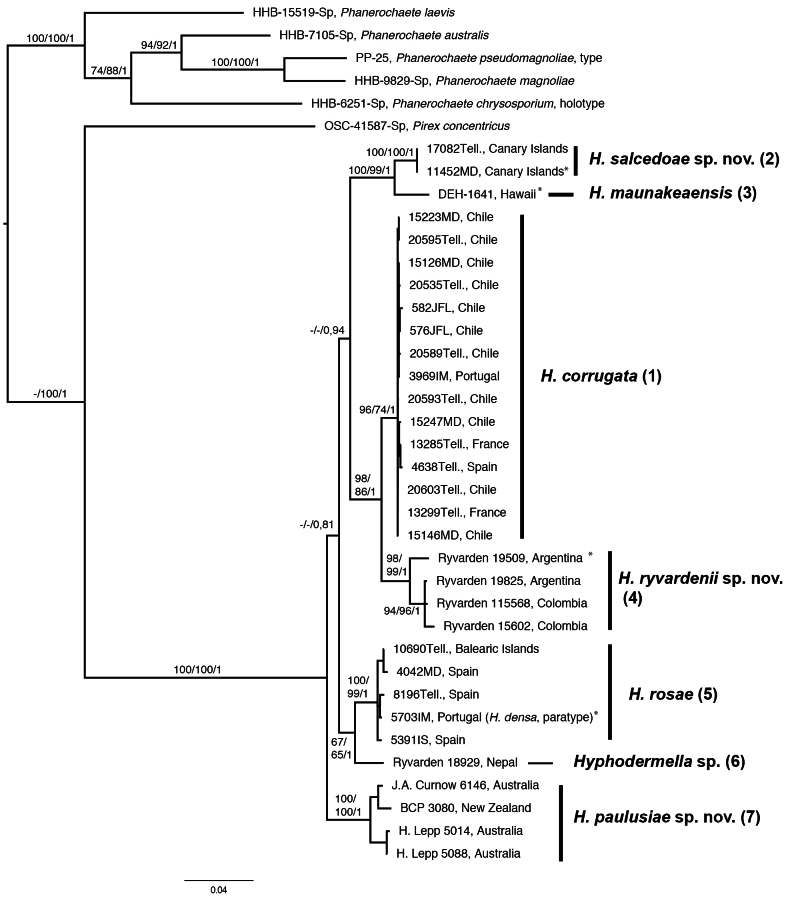
The 50% majority-rule consensus tree from a Bayesian analysis based on the concatenated dataset of ITS nrDNA, LSU nrDNA, *RPB2*, and *RPB1* of *Hyphodermella* species. Five *Phanerochaete* species and *Pirex concentricus* were used as outgroups. Maximum parsimony bootstrap percentages, maximum likelihood bootstrap percentages, and posterior probabilities are indicated at the branches. Type sequences are marked with *.

Dataset 1 (ITS + LSU) consisted of 119 taxa and 1,316 positions, dataset 2 (*RPB2* + *RPB1*) consisted of 45 taxa and 2,020 positions, and dataset 3 (ITS + LSU + *RPB2* + *RPB1*) consisted of 38 taxa and 3,222 positions. Bayesian phylogenies for each combined dataset, with MP bootstrap support (MPbs), ML bootstrap support (MLbs), and posterior probability (PP) values indicated on the branches, are shown in Figs 2, 3, 4.

In Fig. 2 (dataset 1: ITS + LSU), the monophyly of *Hyphodermella* was well supported (MPbs = 100%, MLbs = 99%, PP = 1.0), excluding the four species mentioned above. *Hyphodermella* specimens were distributed among four clades and two singletons, numbered one to six in Fig. 2 (top to bottom), and a separate group comprising Australian and New Zealand specimens that did not form a unique clade (number seven in Fig. 2). Clade 1, strongly supported (MPbs = 94%, MLbs = 88%, PP = 0.99), included *H. corrugata* specimens from Telleria et al. (2010) collected in Portugal, France, Spain, and Morocco, as well as 57 *Hyphodermella* specimens from Chile. Clade 2, weakly supported, comprised two specimens from El Hierro (Canary Islands). Singleton 3 corresponded to the paratype of *H. maunakeaensis* (DEH1641) from Hawaii. Clade 4, strongly supported (MPbs = 96%, MLbs = 96%, PP = 1.0), comprised three specimens from Argentina and Colombia. Clade 5 grouped *H. rosae* (MPbs = 55%, MLbs = 60%, PP = 1.0) from Telleria et al. (2010), collected in Portugal and Spain, with new GenBank sequences obtained from specimen FP 150552 (Hawaii) and isolate LSPQ-NSM-019 (Canada). Finally, singleton 6 corresponded to specimen Ryvarden 18929 from Nepal. In addition, as indicated in Table 1, one specimen from South Korea (KUC11033), for which only an ITS sequence was obtained, and nine GenBank isolates from Azerbaijan, France, Iran, and the USA, represented only by ITS or LSU sequences, clustered within the *H. rosae* group in individual-marker analyses (data not shown).

In Fig. 3 (dataset 2: *RPB2* + *RPB1*), the monophyly of the genus *Hyphodermella* is highly supported (MPbs = 100%, MLbs = 100%, PP = 1), and similar terminal branches are obtained as in the combined ITS + LSU analyses: Clade (2) and Clade (5), both highly supported, as well as singleton (3) and singleton (6). It is important to note that isolates from Australia and New Zealand form a unique Clade (7), which is highly supported (MPbs = 100%, MLbs = 100%, PP = 1). In Clade (1), *H. corrugata*, two specimens from Argentina and one from Colombia differ in their position in the individual marker *RPB2* and *RPB1* analyses, as mentioned above; this explains the subclade formed within Clade (1) of *H. corrugata*, since these specimens form a unique clade in *RPB2* analyses (MPbs = 98%; data not shown), whereas in *RPB1* they are included in the clade of *H. corrugata* (data not shown). On the other hand, the lectotype of *H. ochracea*, from which neither ITS nor LSU sequences were obtained, has *RPB2* and *RPB1* identical to *H. corrugata* specimens and, in both individual marker analyses, is grouped in the clade of this species.

In Fig. 4 (Dataset 3: ITS + LSU + *RPB2* + *RPB1*), the monophyly of the genus *Hyphodermella* is also highly supported (MPbs = 100%, MLbs = 100%, PP = 1); five clades and two singletons can be distinguished, and relationships among clades are better supported than in analyses of datasets 1 and 2.

Clade (1), with strong support (MPbs = 96%, MLbs = 74%, PP = 1), includes *H. corrugata* specimens, which are the sister-group of Ryvarden’s Argentina and Colombia specimens that form a unique Clade (4), highly supported (MPbs = 98%, MLbs = 99%, PP = 1), which we described here as *H. ryvardenii***sp. nov**. This relationship is also highly supported (MPbs = 98%, MLbs = 86%, PP = 1).

Clade (2) contains two specimens from El Hierro (Canary Islands), highly supported (MPbs = 100%, MLbs = 100%, PP = 1), which are a sister-group of singleton (3), *H. maunakeaensis* from Hawaii, which we describe here as *H. salcedoae***sp. nov**. This relationship is highly supported (MPbs = 100%, MLbs = 99%, PP = 1).

Clade (5), highly supported (MPbs = 100%, MLbs = 99%, PP = 1), includes specimens of *H. rosae* from Portugal, Spain (including the Balearic Islands), and Morocco, as a sister-group of singleton (6) from Nepal. The relationship is highly supported in the Bayesian analysis (PP = 1).

Clade (7) comprises specimens from Australia and New Zealand, with high support (MPbs = 100%, MLbs = 100%, PP = 1), which we describe here as *H. paulusiae***sp. nov**. In the phylogenetic tree, this clade (7) is the sister-group of the *Hyphodermella* taxa.

### Morphological and statistical analyses

All species delimited in the seven lineages resulting from the phylogenetic analysis share the characters of the original description of the genus: “basidioma resupinate and effuse, hymenophore irregularly odontioid; hyphal system monomitic, with hyphae hyaline, non-fibulatae; without cystidia, but with encrusted cystidioid hyphae forming fascicles that project in the hymenium; basidia clavate and spores ellipsoid, smooth, thin-walled, and non-amyloid.”

**Figure 5.**
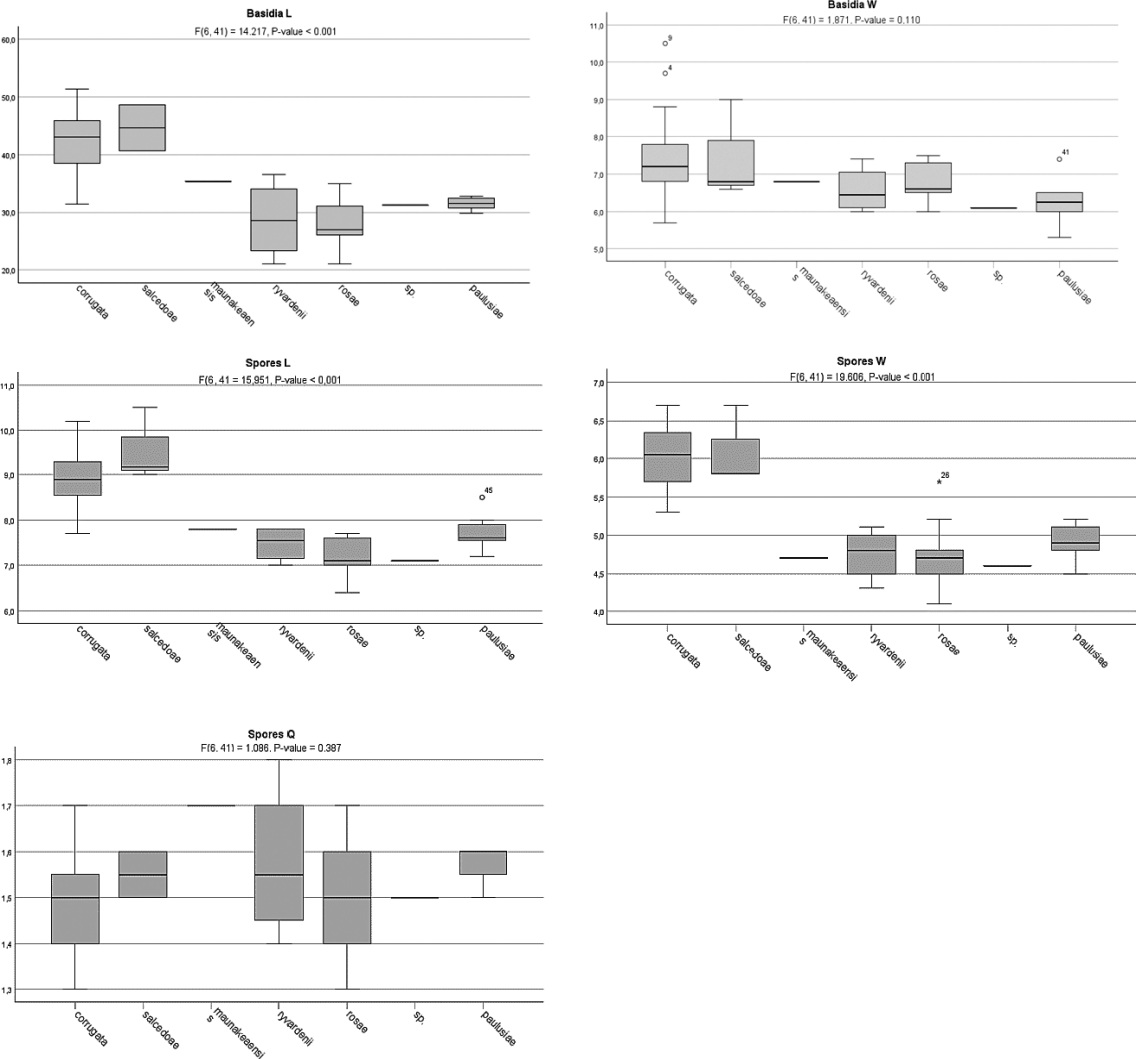
ANOVA results for basidia and basidiospore morphology. Bar plots of *Hyphodermella* species. Length (L), width (W), and length/width ratios (Q). Graphs were generated using IBM SPSS Statistics.

The ANOVA analysis revealed significant differences among the clades (Table 3, Fig. 5) in terms of basidia length (F(6, 41) = 14.217, P-value < 0.001), spore length (F(6, 41) = 15.951, P-value < 0.001), and spore width (F(6, 41) = 19.606, P-value < 0.001), but did not detect statistically significant differences in basidia width (F(6, 41) = 1.871, P-value = 0.110) and the spore Q value (F(6, 41) = 1.086, P-value = 0.387). In the Tukey HSD post hoc test, two groups are formed in the analysis of the length of basidia and the length and width of the spores, one with *H. corrugata* (Clade 1) and *H. salcedoae* (Clade 2), with basidia and spores larger than the others formed by *H. ryvardenii* (Clade 4), *H. rosae* (Clade 5), and *H. paulusiae* (Clade 7). However, as in Telleria et al. (2010), the morphological delimitation between species is ambiguous, with overlapping basidia and spore size in collections of different species, but the mean length of basidia and spores of all collections studied can be considered a useful character to delimit the species.

**Table 3.** Statistical tests of morphological characters. ANOVA of basidia and spore morphology.

	F(6, 41)	*P*-value
Basidia length	14.217	< .001
Basidia width	1.871	.110
Spores length	15.951	< .001
Spores width	19.606	< .001
Spores length/with	1.469	.212

### Taxonomy

Based on the results of the morphological and phylogenetic analyses, we recognize five species in *Hyphodermella* and provide descriptions of three new species. A key with the species recognized is also included.

#### Key to the species in *Hyphodermella*

**Table:** 

1	Basidioma cream to orange yellow when dried, turning red wine with KOH; margin membranaceous, white	***H. ryvardenii***
–	Basidioma not changing color with KOH; margin non-membranaceous	**2**
2	Mean length of basidia > 40 (basidia 30–70 × 5–11 μm), and mean length of spores > 8 (spores 7–11 × 5–8 μm)	**3**
–	Mean length of basidia < 40 (basidia 21–42(–46) × 5–9 μm) and mean length of spores < 8 (spores 5.5–9(–10) × (3.5–)4–6 μm)	**4**
3	Basidioma orange to brown when dried; odontioid, with scattered to crowded aculei; encrusted cystidioid hyphae abundant; on a wide variety of substrates	***H. corrugata***
–	Basidioma cream to pale yellow when dried; almost smooth; encrusted cystidioid hyphae scattered, sometimes difficult to see; known so far only in the Canary Islands, on *Chamaecytisus proliferus*	***H. salcedoae***
4	Basidioma cream to pale yellow when dried; subicular hyphae densely interwoven, sometimes forming a pseudoparenchymatic texture; mean length of basidia < 30 and mean length of spores < 7.5	***H. rosae***
–	Subicular hyphae not forming a pseudoparenchymatic texture basidia and spores longer	**5**
5	Basidioma cream to yellow grayish, thin at first, thicker and stratified with age: hymenophore odontioid, with scattered to crowded aculei; mean basidia 31.5 × 6.3; mean spores 7.7 × 4.9; known from Australia and New Zealand	***H. paulusiae***
–	Basidioma cream to pale yellow when dried; thin, hymenophore hydnoid; mean basidia 31.2 × 6.8; mean spores 7.8 × 4.7; known only from Hawaii	***H. maunakeaensis***

##### Hyphodermella corrugata

Taxon classificationFungiPolyporalesCorticiaceae

(Fr.) J. Erikss. & Ryvarden, Corticiaceae North Europe 4: 579. 1976

3CE69151-C5E1-5683-AFDF-9E0252A74439

Fig. 6A

≡ Grandinia corrugata Fr., Hymenomycetes Europaei: 625. 1874
= Hyphodermella ochracea (Bres.) Duhem, Bull. Soc. Mycol. France 125 (3, 4): 158. 2010 [2009] ≡Epithele ochracea Bres., Ann. Mycol. 18(1–3): 48 (1920). MycoBank No: 272673. Type: Italy. Tridenti (Trento): Ad ramos, truncos *Ampelopsidis [Ampelopsis] hederacea*, 1891, G.Bresadola, Ex Herb. Romell [lectotype: F-89175, S!, designed by Duhem 2010: 158].

###### Type.

Norway • leg. M.N. Blytt, F-128815; UPS 410645.

###### Description and iconography.

In Telleria et al. (2010).

###### Habitat and distribution.

*Hyphodermella corrugata* has been collected on decayed wood, mostly from angiosperms (Telleria et al. 2010). In addition, we have found this species fruiting on *Amomyrtus luma*, *Berberis* sp., *Laureliopsis philippiana*, *Laurus nobilis*, *Lophosoria quadripinnata*, *Luma apiculata*, *Lomatia ferruginea*, *Nothofagus dombeyi*, and *Rubus* sp. Widely distributed in the Northern Hemisphere, it is known from the Southern Hemisphere from the Patagonian Andes of Argentina (Gorjón et al. 2012); we confirm its presence and abundance in Chile, where it was reported by Ortiz et al. (2014) from Chiloe. Some species described by Rick (1959a, b) from Brazil as *Cystidiodendron laetum*, *C. papilliforme*, *Hydnochaete laeta*, *Neokneiffia sulphurella*, *Odontia fibrosissima*, *O. horridissima*, *O. subferruginea*, *Radulochaete flavoalutacea*, *Radulum griseum*, *R. subsulphureum*, *R. tenue* have been synonymized to *H. corrugata* (Hjortstam and Ryvarden 1982; Baltazar et al. 2016), but we have not had the opportunity to study them. Two specimens from the Southern Hemisphere, one from Brazil (Tatiana B. Gibertoni N 171, O-917953) and another from Zimbabwe (L. Ryvarden 27245, O-903662), labeled as *H. corrugata*, correspond to *Resinicium* sp. and *Botryodontia denticulata* Hjortstam, respectively.

**Figure 6.**
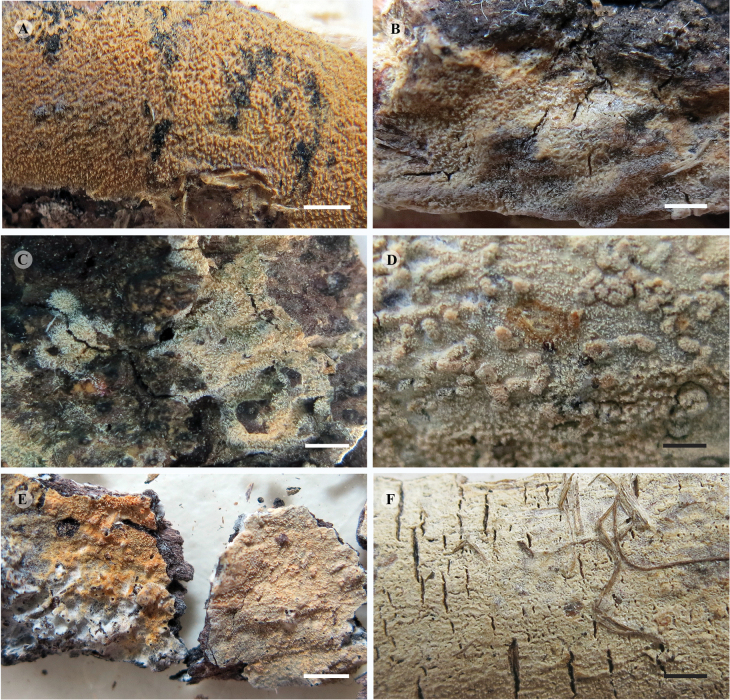
Basidioma of *Hyphodermella* species. **A**. *H. corrugata* (14453MD, MA-Fungi 94244); **B**. *H. maunakeaensis* (DEH-1641, CFMR); **C**. *H. paulusiae* (BCP 4176, PDD 92259); **D**. *H. rosae* (10690Tell., MA-Fungi 36972); **E**. *H. ryvardenii* (L. Ryvarden 19509, F-503386); **F**. *H. salcedoae* (11452MD, MA-Fungi 92643). Scale bars: 2 mm.

###### Specimens examined.

Chile - **Los Lagos**: Palena, Comuna Hualaihué, Huinay Biological Reserve, “Cementerio de los Alerces” experimental plot; 42°21'57.9"S, 72°24'56.9"W; 30 m alt.; 29 Apr 2012; on *Amomyrtus luma*; M.T.Telleria leg.; 19693Tell., MA-Fungi 94278 • same data as for preceding, 19696Tell., MA-Fungi 94279 • ibid; 42°22'01.5"S, 72°24'57.8"W; 50 m alt.; 10 May 2013; on unidentified wood; M.Dueñas leg.; 14646MD, MA-Fungi 94248 • Palena, Comuna Hualaihué, Huinay Biological Reserve, path to Lloncochaigua river; 42°22'27.3"S, 72°24'39.5"W; 178 m alt.; 5 May 2013; on *Lophosoria quadripinnata*; M.T.Telleria leg.; 20022Tell., MA-Fungi 94285 • same data as for preceding, 20023Tell., MA-Fungi 94286 • Palena, Comuna Hualaihué, Huinay Biological Reserve, near to “Derrumbe Antiguo”; 42°22'11.9"S, 72°24'18.1"W; 54 m alt.; 6 May 2013; on unidentified wood, M.T.Telleria leg.; 20041Tell., MA-Fungi 94287 • Palena, Comuna Hualaihué, Huinay Biological Reserve, suspension bridge path; 42°22'09.0"S, 72°24'42.7"W; 19 m alt.; 30 Apr 2012; on *Luma apiculata*; M.T.Telleria leg.; 19704Tell., MA-Fungi 94280 • ibid; 42°22'38.9"S, 72°24'45.8"W; 190 m alt.; 4 May 2013; on unidentified wood; M.Dueñas leg.; 14453MD, MA-Fungi 94244 • same data as for preceding, 14454MD, MA-Fungi 94245 • same data as for preceding, 14458MD, MA-Fungi 94246 • ibid; M.P. Martín leg.; 3492MPM, MA-Fungi 94275 • ibid; on *Amomyrtus luma*; M.Dueñas leg.; 14462MD, MA-Fungi 94247 • ibid; on *Lomatia ferruginea*; M.P.Martín leg.; 3484MPM, MA-Fungi 94274 • ibid; 42°22'27.3"S, 72°24'39.5"W; 178 m alt.; 5 May 2013; on fallen branches; M.P.Martín leg.; 3498MPM, MA-Fungi 94276 • same data as for preceding, 3511MPM, MA-Fungi 94277 • ibid; 42°22'07.7"S, 72°24'16.1"W; 21 m alt.; 19 Oct 2014; on unidentified wood; M.Dueñas and M.T. Telleria legs.; 15222MD, MA-Fungi 94265 • same data as for preceding,15223MD, MA-Fungi 94266 • same data as for preceding, 15232MD, MA-Fungi 94267 • same data as for preceding, 15233MD, MA-Fungi 94268; 15240MD, MA-Fungi 94269 • ibid; 20588Tell., MA-Fungi 94292 • same data as for preceding, 20589Tell., MA-Fungi 94293 • same data as for preceding, 20591Tell., MA-Fungi 94294 • same data as for preceding, 20592Tell., MA-Fungi 94295 • same data as for preceding, 20603Tell., MA-Fungi 94302 • same data as for preceding, 20604Tell., MA-Fungi 94303 • same data as for preceding, 20605Tell., MA-Fungi 94304 • same data as for preceding, 20607Tell., MA-Fungi 94305 • same data as for preceding, 20608Tell., MA-Fungi 94306 • ibid; on *Luma apiculata ; 20593Tell*., MA-Fungi 94296 • same data as for preceding, 20595Tell., MA-Fungi 94297 • same data as for preceding, 20597Tell., MA-Fungi 94298 • same data as for preceding, 20598Tell., MA-Fungi 94299 • same data as for preceding, 20599Tell., MA-Fungi 94300 • same data as for preceding, 20600Tell., MA-Fungi 94301 • same data as for preceding, 20603Tell., MA-Fungi 94302 • same data as for preceding, M. Dueñas leg.; 15243MD, MA-Fungi 94270 • same data as for preceding, 15247MD, MA-Fungi 94271 • ibid; path to hydroelectric station; 42°23'04.3"S, 72°25'47.3"W; 3 May 2013; on unidentified wood; M.T.Telleria leg.; 19949Tell.; MA-Fungi 94281 • same data as for preceding,19951Tell., MA-Fungi 94385; 19957Tell., MA-Fungi 94282 • same data as for preceding, 19963Tell., MA-Fungi 94283 • same data as for preceding, 19967Tell., MA-Fungi 94284 • ibid; near to “Bosque de las Catedrales“; 42°22'23.7"S, 72°24'20.0"W; 70 m alt.; 15 Oct 2014; on fallen branches; M.Dueñas and M.T. Telleria legs.; 15105MD; MA-Fungi 94249 • same data as for preceding, 15106MD, MA-Fungi 94250 • ibid; on *Rubus* sp.; M.Dueñas and M.T. Telleria legs.; 20472Tell., MA-Fungi 94288 • ibid; path to the bridge, under the waterfalls; 42°22'29.6"S, 72°24'42.8"W; 16 Oct 2014; on *Luma apiculata*; M.Dueñas and M.T. Telleria legs.; 15118MD, MA-Fungi 94251 • same data as for preceding, 15121MD, MA-Fungi 94252 • same data as for preceding, 15123MD, MA-Fungi 94253 • same data as for preceding, 15124MD, MA-Fungi 94254 • ibid; on *Berberis* sp.; 15125MD, MA-Fungi 94255 • ibid; on fallen branches; 15126MD, MA-Fungi 94256 • ibid; on unidentified wood; 15146MD, MA-Fungi 94257 • same data as for preceding, 15147MD, MA-Fungi 94258 • same data as for preceding, 15148MD, MA-Fungi 94259 • same data as for preceding, 15149MD, MA-Fungi 94260 • ibid; waterfalls path; 42°22'35.1"S, 72°24'32.8"W; 53 m alt.; 17 Oct 2014; on unidentified wood; M.Dueñas and M.T.Telleria legs.: 20525Tell., MA-Fungi 94289 • same data as for preceding, 20530Tell., MA-Fungi 94290 • ibid; on *Berberis buxicola*; M.Dueñas and M.T.Telleria legs.; 20535Tell., MA-Fungi 94291 • ibid; 18 Oct 2014; on *Laureliopsis philippiana*; M.Dueñas and M.T.Telleria legs.; 15186MD, MA-Fungi 94261 • ibid; on *Luma apiculata*; M.Dueñas and M.T.Telleria legs.; 15194MD, MA-Fungi 94262 • same data as for preceding, 15213MD, MA-Fungi 94263 • same data as for preceding, 15217MD, MA-Fungi 94264 • Palena, Comuna de Palena, highway CH235, Santa Lucía to Puerto Ramírez, km 8, near to “Puente Verde 1”; 43°23'11.1"S, 72°17'54.8"W; 117 m alt.; 31 Oct 2017; on unidentified wood; M.Dueñas leg.; 15499MD, MA-Fungi 98676 • ibid, M.T.Telleria leg.; 20991Tell., MA-Fungi 98677 • ibid; on *Nothofagus dombeyi*; M.Dueñas leg.; 15509MD, MA-Fungi 98678 • Palena, Comuna de Chaitén, Corcovado National Park, highway 7, Austral road, Puerto Cárdenas to Villa Vanguardia, beside Piedras Blancas bridge; 43°36'27.1"S, 72°20'20.6"W; 132 m alt., 31 Oct 2017; on a climbing plant, M.Dueñas leg.; 15485MD, MA-Fungi 98675. • **Los Ríos**: Valdivia, Comuna de Paillaco, highway T-60, km 16; 40°01'08.3"S, 73°08'10.2"W; 130 m alt.; 8 Nov 2017; on unidentified wood; J.Fernández López leg.; 582JFL, MA-Fungi 94273 • same data as for preceding, 576JFL, MA-Fungi 94272. • **Aysén del General Carlos Ibáñez del Campo**: Comuna de Cisnes, highway 7, Austral road, Queulat National Park, beside Queulat bridge; 44°33'52.2"S, 72°27'33.9"W; 38 m alt.; 2 Nov 2017; on unidentified wood; M.Dueñas leg.; 15544MD, MA-Fungi 98679.

##### Hyphodermella maunakeaensis

Taxon classificationFungiPolyporalesPhanerochaetaceae

Gilb. & Hemmes, in Gilbertson, Desjardin, Rogers & Hemmes, Fungal Diversity 6: 53 (2001)

686F390C-D35B-5387-BAEC-6A27AA56F580

Figs 6B, 7

###### Type.

United States • **Hawaii**: Pu’u La’au, Mauna Kea, North Hilo District, Hawai’i Cou.; on *Myoporum sandwicense*; 22 Nov 1998; R.L. Gilbertson 22335 (holotype, BPI).

###### Description and iconography.

In Gilbertson et al. (2001).

###### Habitat and distribution.

On *Myoporum sandwicense*, an endemic species from Hawaii. Species known only from Hawaii, where, according to Gilbertson et al. (2001), it is well represented.

###### Specimen examined.

United States • **Hawaii**, Island of Hawaii, Mauna Kea, Parker PTA; 16 Dec 1997; on naio (*Myoporum sandwicense*); D.E. Hemmes, DEH-1641 (paratype, CFMR!).

**Figure 7.**
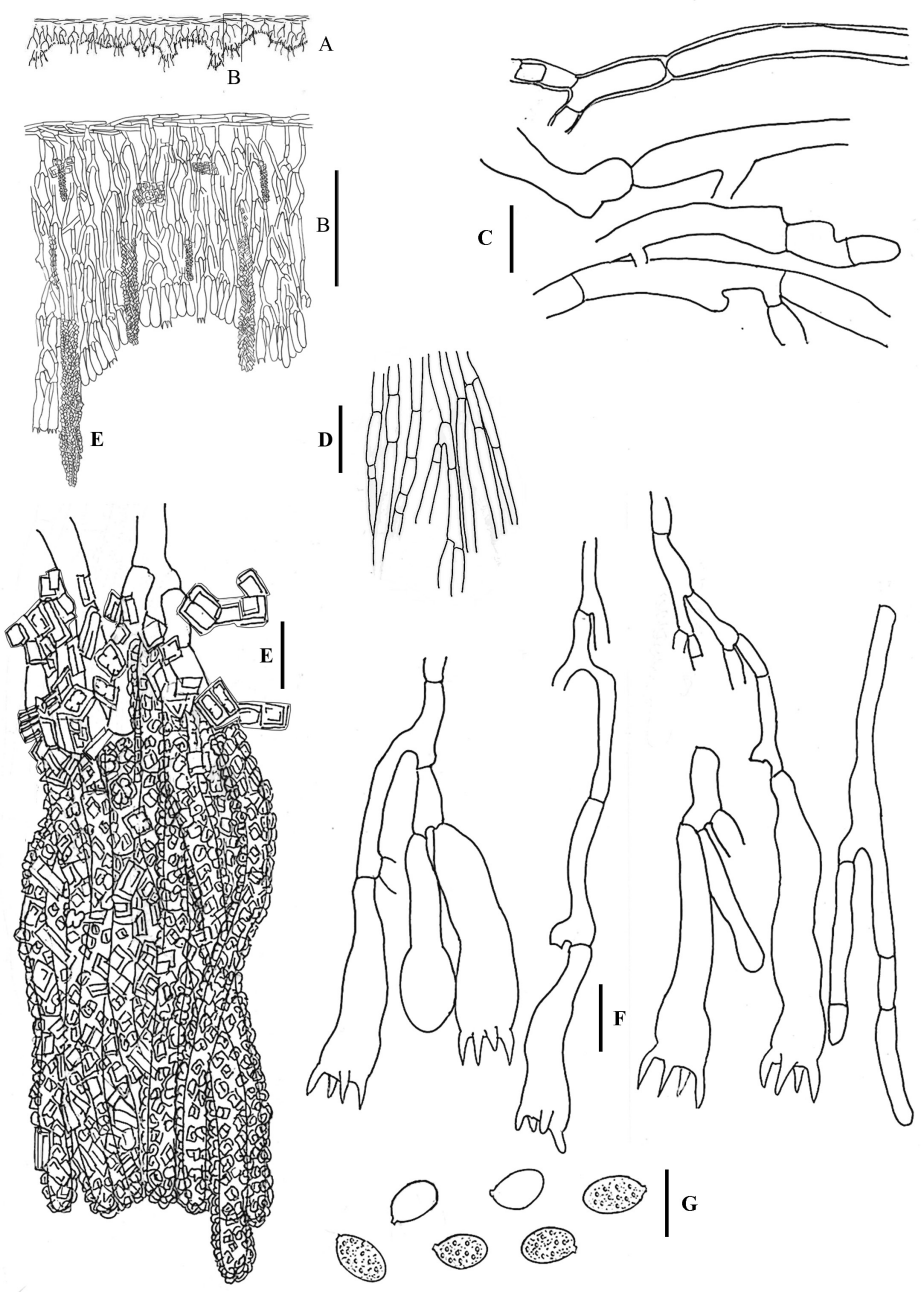
*Hyphodermella maunakeaensis* DEH-1641, CFMR. **A**. Schematic section through the basidioma; **B**. Vertical section of the basidioma; **C**. Subicular hyphae; **D**. Subhymenial hyphae; **E**. Encrusted cystidioid hyphae; **F**. Basidia; **G**. Spores. Scale bars: 100 µm (**B**); 10 µm (**C–G**).

###### Notes.

Our phylogenetic analyses (Figs 1, 2, 3, 4) support the identity of *H. maunakeaensis*. Gilbertson et al. (2001) described *H. maunakeaensis* with basidia 15–25 × 5–7 μm and spores 6.5–7.5 × 3.5–4 μm, smaller than the specimen studied, 26–37 × 6–7 μm and 6.5–9 × 4.5–5 μm respectively, Q = 1.7, but these data must be taken with caution, since we have only studied one specimen. Hjortstam and Ryvarden (2007) reported one specimen from Venezuela as *H. aff. maunakeaensis*, we have not been able to locate this material, and it would be interesting to review it to better understand the distribution of this species.

##### Hyphodermella paulusiae

Taxon classificationFungiPolyporalesPhanerochaetaceae

M. Dueñas, Telleria & M.P. Martín sp. nov.

5D9BFDBA-8022-555A-823D-B674332FD4BC

MB860663

Figs 6C, 8

###### Type.

New Zealand • **Bay of Plenty**: near Ruatahuna, Tarapounamu, on decaying branch; 3 Dec 2006; B.C.Paulus and P.R.Johnston legs.; BCP 4176, (holotype 92259, PDD!, GenBank: MZ147703, MZ147778, MZ147915).

**Figure 8.**
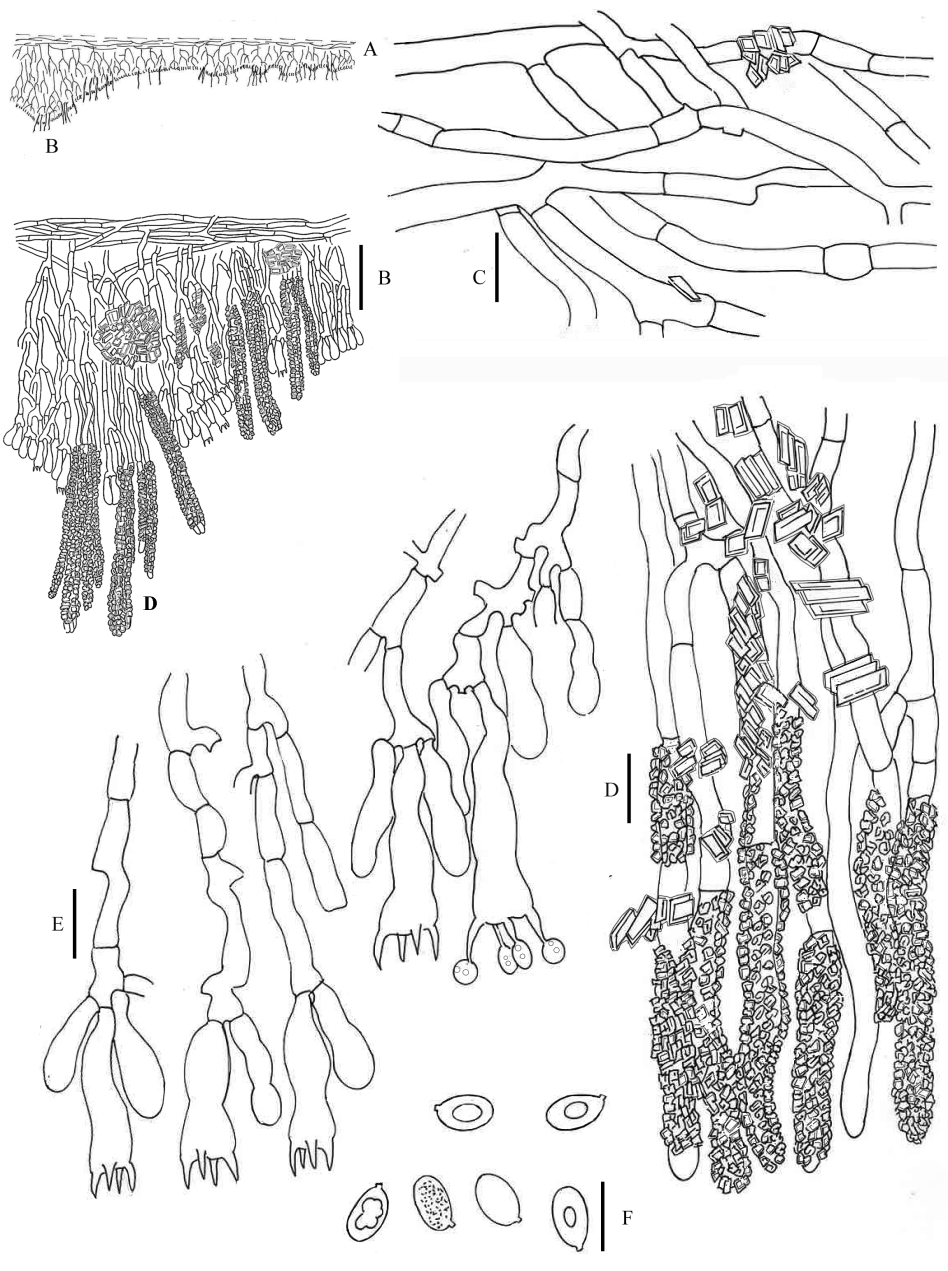
*Hyphodermella paulusiae* BCP 4176, PDD 92259. **A**. Schematic section through the basidioma; **B**. Vertical section of the basidioma; **C**. Subicular hyphae; **D**. Subhymenial hyphae; **E**. Encrusted cystidioid hyphae; **F**. Basidia; **G**. Spores. Scale bars: 100 µm (**B**); 10 µm (**C–G**).

###### Etymology.

Named after Barbara Paulus, a New Zealand mycologist, who collected the holotype and for her significant contribution to the knowledge of New Zealand's mycobiota.

###### Diagnosis.

Macroscopically this new species is similar to *H. rosae*; it is characterized by the size of basidia, 25–42 × 5–8 μm, L = 31.5, W = 6.3, spores 6–10 × 4–6 μm, L = 7.7, W = 4.9, and by the phylogenetic position as sister of the other *Hyphodermella* species.

###### Description.

**Basidioma** resupinate, adnate, orbicular to confluent, thin at first, thicker and stratified with age, crustose; hymenophore ceraceous, cream (89.p. Y), yellow grayish (90. gy. Y), darker with age (91. d. gy. Y – 87. m. Y), odontioid, with scattered to crowded aculei, under the lens penicillate by the projection of encrusted cystidioid hyphae, cracked with age; margin fibrillose when young, determinate with age, lighter than the hymenophore. **Hyphal system** monomitic; hyphae hyaline, without clamps, distinct, thin- to thick-walled, (2–)3–5 μm, sometimes encrusted; numerous encrusted cystidioid hyphae forming fascicles, projecting in the hymenium. **Basidia** clavate, with constrictions, 25–42 × 5–8 μm, L = 31.5, W = 6.3, with 4 sterigmata. **Spores** ellipsoid, with oil drops in the protoplasm, 6–10 × 4–6 μm, hyaline, thin-walled and smooth. L = 7.7, W = 4.9, Q = 1.6.

###### Habitat and distribution.

On decayed and rotten wood in *Callitris*, *Eucalyptus*, and *Nothofagus* forests. Known from Australia and New Zealand.

###### Additional specimens examined.

Australia • **New South Wales**: North Western Plains, Killarney State Forest, 16.6 km (by road) N of Narrabri, Spargo Road of Murrumbilla Lane; 30°13'19"S, 149°50'47"E; 270 m alt.; on rotten wood on *Eucalyptus*–*Callitris* forest; 11 May 2005; H.Lepp 5014, CANB 752189 • **North Western Slopes**, Warrumbungle National Park, Whitegum Lookout carpark, 24 km (by road) W of Coonabarabran; 31°17'22"S, 149°02'29"E; 720 m alt.; on fallen, rotting, eucalypt branch; 12 May 2005, H.Lepp 5088 • North Coast, Cottan-Bimbang National Park, Myrtle Gully Road / Scenic Drive, 4.8 km along road (from Walcha end) from Osley Highway; 31°21'45"S, 152°00'38"E; 980 m alt.; on soft dead stem loose on ground in rainforest with isolated eucalypts and *Nothofagus*; 28 Apr 2005; J.A.Curnow 6146, CANB 889543. New Zealand • **Te Urewera**: Mangapae UA1 E, n.m, on decaying wood; 11 Oct 2004; P.R.Johnston and B.C.Paulus legs.; BCP 3073, PDD 94113 • ibid; BCP 3080, PDD 94114 • ibid; Tarapounamu, W side Rd.; 27 Oct 2009; P.R. Johnston and B.C. Paulus; PRJ47, PDD 85550.

##### Hyphodermella rosae

Taxon classificationFungiPolyporalesPhanerochaetaceae

(Bres.) Nakasone, Cryptogamie Mycologie 29: 251. 2008

F2E8169E-D1AF-592E-9108-98940B037C88

Fig. 6

###### Type.

Italy • prope Trento, ad ramos *Rosa* sp., Dec 1924, leg. Remis [holotype, TR B/3785]

###### Description and iconography.

In Telleria et al. (2010).

###### Habitat and distribution.

*Hyphodermella rosae* fruits on decayed wood of various angiosperm species, both trees and shrubs (Telleria et al. 2010), also have been reported as phytopathogens (Babaeizad et al. 2012; Rahimlou et al. 2015; Dell’Ollmo et al. 2024). It is widely distributed in the Northern Hemisphere, confirming the idea suggested by Telleria et al. (2010) that *H. rosae* has a distribution area as wide as *H. corrugata*.

##### Hyphodermella ryvardenii

Taxon classificationFungiPolyporalesPhanerochaetaceae

M. Dueñas, Telleria & M.P. Martín sp. nov

F6CD5733-1CFE-549D-B234-739D39B89D55

MB860665

Figs 6E, 9

###### Type.

Argentina – **Misiones** • Iguazú Nat. Park, Cataratas de Iguazú; 1–5 Mar 1982; on deciduous wood; L.Ryvarden 19509 (holotype F-503386, O!, GenBank: MZ147710, MZ147772, MZ147912, MZ147958).

**Figure 9.**
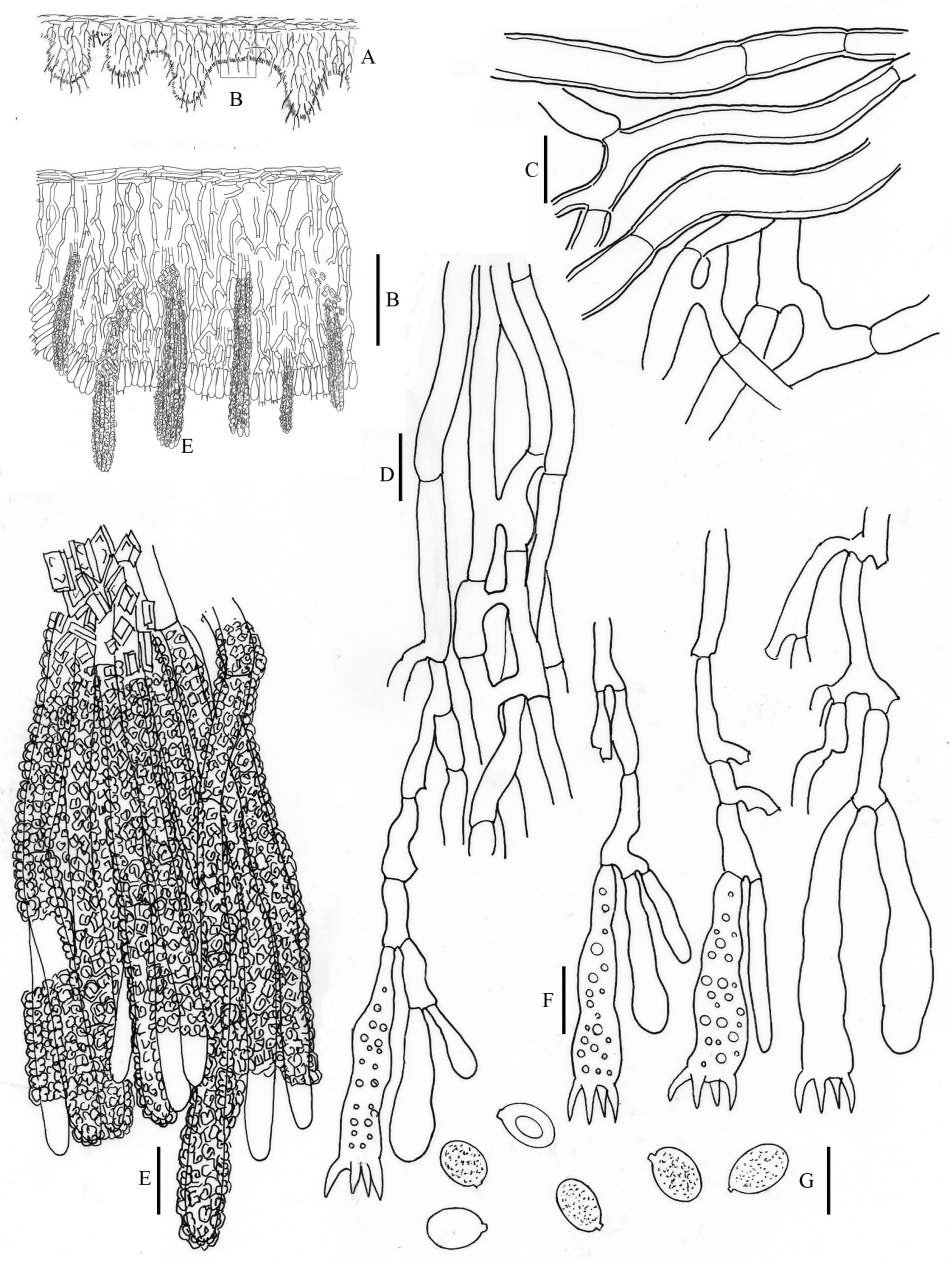
*Hyphodermella ryvardenii* L. Ryvarden 19509, F-503386. **A**. Schematic section through the basidioma; **B**. Vertical section of the basidioma; **C**. Subicular hyphae; **D**. Subhymenial hyphae; **E**. Encrusted cystidioid hyphae; **F**. Basidia; **G**. Spores. Scale bars: 100 µm (**B**); 10 µm (**C–G**).

###### Etymology.

Named after Leif Ryvarden, Norwegian mycologist, for his contribution to the understanding of the diversity of wood-inhabiting fungi worldwide, particularly in Europe, Africa and tropical America, who also collected all the specimens of this new species.

###### Diagnosis.

Macroscopically, this species is similar to *H. corrugata* but differs in the basidioma that turns wine-red with KOH, in the membranaceous subiculum, and in the smaller size of the basidia, 21–41(–46) × 5.5–9 μm, L = 28.7, W = 6.6 μm, and spores, 7–9 × 4–6 μm, L = 7.5, W = 4.8 μm, Q = 1.6.

###### Description.

**Basidioma** resupinate, adnate, orbicular to confluent, thin, thickening with age, crustose; subiculum membranaceous, white; hymenophore ceraceous, at first white, with age cream (92. y White) to orange yellow (69. deep OY – 72. d. OY), turning wine red in KOH 5%, odontioid, with scattered to crowded aculei, under the lens penicillated by the projection of encrusted cystidioid hyphae; margin at first membranaceous, white, determined with age. **Hyphal system** monomitic, hyphae hyaline, without clamps; subicular hyphae distinct, thin- to thick-walled, 3.5–7 μm wide; subhymenial hyphae thin-walled, 2–4 μm wide. Numerous encrusted cystidioid hyphae forming fascicles projecting in the hymenium. **Basidia** clavate, with constrictions, 21–41(–46) × 5.5–9 μm, L = 28.7, W = 6.6, with 4 sterigmata up to 7 μm long. **Spores** ellipsoid, with oil drops in the protoplasm, 7–9 × 4–6 μm, hyaline, thin-walled, and smooth. L = 7.5, W = 4.8, Q = 1.6.

###### Habitat and distribution.

On decayed wood. Known from Colombia and Iguazú National Park from Argentina.

###### Additional specimens examined.

Argentina – **Misiones** • Iguazú Nat. Park, Cataratas de Iguazú; 1–5 Mar 1982; on deciduous wood; L. Ryvarden 19825, F-503387 (O). Colombia • Dpto. de Cundinamarca, km 20 en la vía Mosquera-La Mesa; 2300 m alt.; 6 Jun 1978: L. Ryvarden 15568, F-918413 (O) • same data as for preceding L. Ryvarden 15602, F-918415 (O).

###### Notes.

This new species is phylogenetically closely related to *H. corrugata*; in fact, the combined analysis of *RPB1* and *RPB2* (Fig. 3) does not discriminate between the two species but in the concatenated analysis of four markers, these specimens form a highly supported clade (Fig. 4). Morphologically, they can be separated by the change in color to wine-red with KOH in H. ryvardenii and by the mean values of basidia, 28.7 × 6.6, and spores, 7.5 × 4.8, Q = 1.6 in *H. ryvardenii*, instead of 42.4 × 7.4 μm and 8.9 × 6.0, Q = 1.5 in *H. corrugata*.

##### Hyphodermella salcedoae

Taxon classificationFungiPolyporalesPhanerochaetaceae

M. Dueñas, Telleria & M.P. Martín sp. nov.

4C9804F3-129F-5D90-9E78-E8AD61B280F4

860669

Figs 6C, 10

###### Type.

Spain – **Canary Islands**, El Hierro: Valverde, road from San Andrés to El Pinar, Los Llanos de San Andrés; 27°45'03"N, 17°58'02"W; 1183 m alt.; 28 Jan 2007; on *Chamaecytisus proliferus*; M.Dueñas leg.; 11452MD (holotype, MA-Fungi 92643, GenBank: MZ147770, MZ147697, MZ147923, MZ147935).

**Figure 10.**
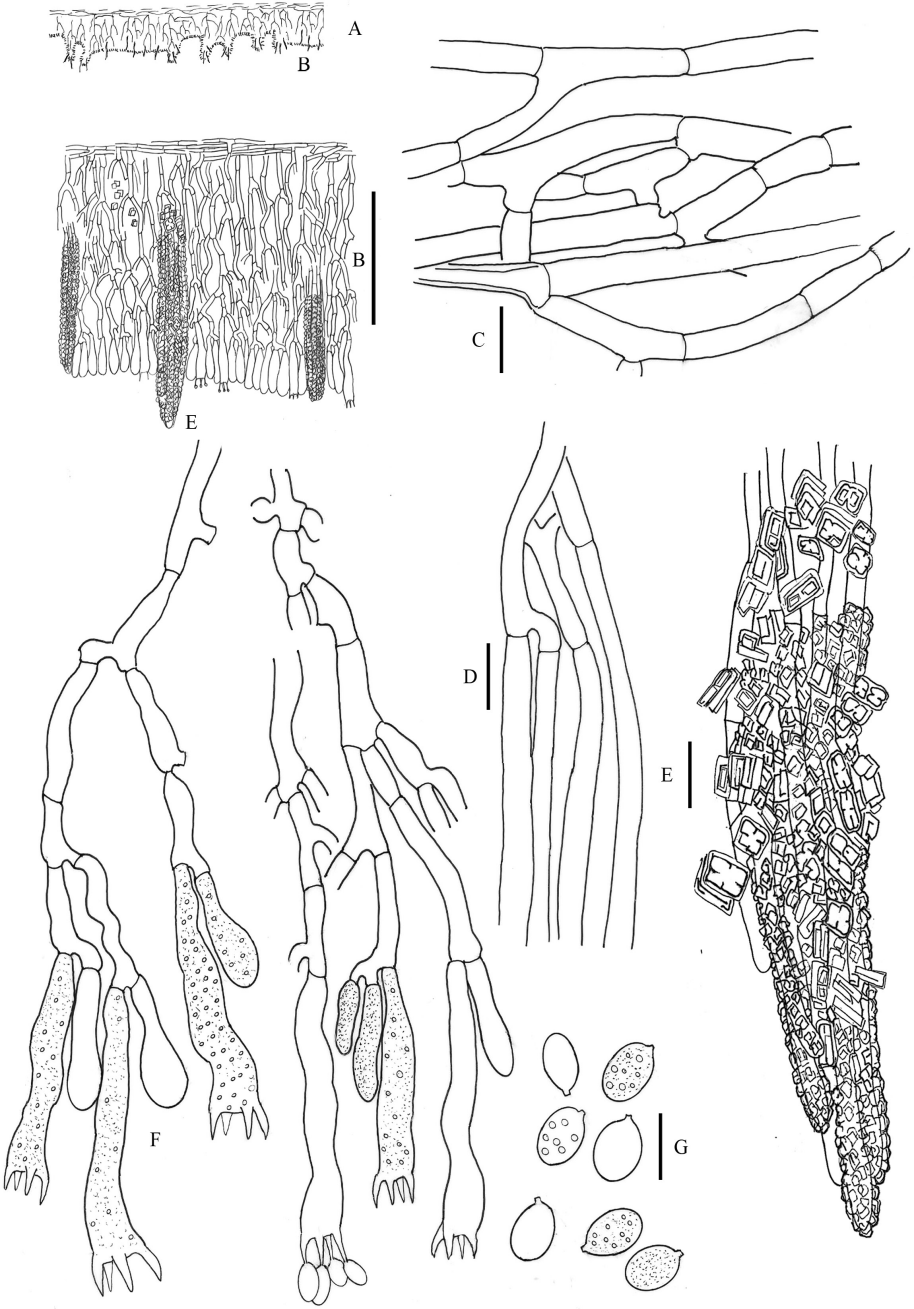
*Hyphodermella salcedoae* 11452MD, MA-Fungi 92643. **A**. Schematic section through the basidioma; **B**. Vertical section of the basidioma; **C**. Subicular hyphae; **D**. Subhymenial hyphae; **E**. Encrusted cystidioid hyphae; **F**. Basidia; **G**. Spores. Scale bars: 100 µm (**B**); 10 µm (**C–G**).

###### Etymology.

Named after Isabel Salcedo, a colleague and friend from the Department of Botany at the University of the Basque Country, with whom we have shared a good number of expeditions.

###### Diagnosis.

This new species is characterized by the hymenophore cream to pale yellow, almost smooth, with scattered aculei; basidia 30–52 × 5–8 μm, L = 44.7, W = 6.6 and spores, 8–10 × 5–7 μm, L = 9.4, W = 6, Q = 1.6.

###### Description.

**Basidioma** resupinate, adnate, thin at first, cracked with age, crustose; hymenophore ceraceous, cream to pale yellow (89.p. Y), almost smooth, with scattered aculei; margin fibrillose, determinate with age, lighter than hymenophore. **Hyphal system** monomitic; hyphae hyaline, without clamps, distinct, thin-walled, 2–5(–8) μm wide; encrusted cystidioid hyphae scattered and sometimes difficult to see. **Basidia** clavate, with constrictions, 30–52 × 5–8 μm, L = 44.7, W = 6.6, with 4 sterigmata. **Spores** ellipsoid, with oil drops in the protoplasm, 8–10 × 5–7 μm, hyaline, thin-walled and smooth. L = 9.4, W = 6, Q = 1.6.

###### Habitat and distribution.

On *Chamaecytisus proliferus*, an endemic species of the Canary Islands. It is only known from the island of El Hierro (Canary Islands), so far.

###### Additional specimen examined.

Spain – **Canary Islands**, El Hierro: Valverde, road from San Andrés to El Pinar, Los Llanos de San Andrés; 27°45'03"N, 17°58'02"W; 1183 m alt.; 28 Jan 2007; on *Ch. proliferus*; M.T.Telleria leg.; 17082Tell., MA-Fungi 92646.

###### Notes.

We initially identified these specimens as a *Phanerochaete* with large spores because the basidiome is almost smooth and the characteristic encrusted cystidioid hyphae are sometimes difficult to see. However, a previous molecular analysis placed them in *Hyphodermella*. Their microscopic characters are similar to *H. corrugata*, but molecular analyses of two (RPB2 + RPB1, Fig. 3) and four markers (ITS + LSU + RPB2 + RPB1, Fig. 4) place them close to *H. maunakeaensis*. Both differ, in the alignment of four markers (ITS + LSU + RPB2 + RPB1), by a total of 66 base pairs and in the morphology of the basidioma, clearly odontioid, with smaller basidia, 26–37 × 6–7 μm, and spores, 6.5–9 × 4.5–5 μm, Q = 1.7, in *H. maunakeaensis*.

## Discussion

As in Telleria et al. (2010), our molecular analyses provided strong support for the monophyly of *Hyphodermella* (Figs 1, 2, 3, 4), unlike Chen et al. (2021), since in their phylogenetic analysis of five markers they considered this genus to be paraphyletic. Our ITS and LSU analyses delimited seven species in the *Hyphodermella* core, and the same species were delimited in our multilocus coalescent approach, confirming that ITS adequately discriminates between species in the genus.

Although ITS is effective for species delimitation in fungi, according to Cao et al. (2021), in Polyporales it is insufficient to delimit some taxa, such as the *Postia caesia* complex (Pildain and Rajchenberg 2013), where the combined analysis of ITS and *tef*1 was able to delimit 20 species in the complex (Miettinen et al. 2018). Justo et al. (2017) recommended that, to obtain robust phylogenies in Polyporales, the combined analysis of ribosomal RNA genes and protein-coding genes, especially *RPB1*, should be included. In our study, the results of the ITS and ITS/LSU analyses were similar to those of the multilocus phylogenetic analysis based on four markers (ITS, LSU, *RPB1*, and *RPB2*) and confirmed that ITS discriminates well among species of the genus; however, *RPB1* and *RPB2* did not discriminate well between *H. ryvardenii* and *H. corrugata* (Fig. 2).

Sequence-based identification methods are powerful tools to delimit fungal species, but the selection of suitable taxa for molecular analysis and rigorous data interpretation is necessary to make accurate inferences, together with phenotype-based verification, ecological strategy, and biogeography (Lücking et al. 2020; Cao et al. 2021). *Hyphodermella aurantiaca*, *H. pallidostraminea*, *H. poroides*, and *H. zixishanensis* were described based on BLAST searches (Zhao et al. 2017; Wang and Zhao 2020; Crous et al. 2021) that placed them close to the *Hyphodermella* core, although diagnostic characters of the genus, such as the odontioid basidiome with penicillate aculei and encrusted cystidioid hyphae, were not described. Shen et al. (2023) gave *Hyphodermella* as an example of studies based on simple phylogenies that do not always place species in appropriate genera; they erected *Pseudohyphodermella* to include *H. poroides* and transferred *H. aurantiaca* and *H. zixishanensis* to *Roseograndinia*. Our molecular analysis of ITS (Fig. 1) confirms, as in Shen et al. (2023), that these species do not belong to *Hyphodermella* and supports the original description of the genus.

The ITS region is the most widely used barcode marker in fungi and has also been successfully applied to sequence historical herbarium specimens. Telleria et al. (2010) obtained the ITS sequence of the type of *H. rosae*, collected in 1924, which allowed its synonymization with *H. densa*. In our study, neither ITS nor LSU sequences were obtained from the type of *H. ochracea* (F-89175), collected in 1891 (Figs 1, 2), but the *RPB1* and *RPB2* sequences were identical to those of *H. corrugata* (Figs 3, 4). This genetic evidence, combined with morphological characteristics, supports the consideration of *H. ochracea* and *H. corrugata* as conspecifics.

As in Telleria et al. (2010), the mean values of basidia and spore size can be considered the most useful characters with high diagnostic value. However, in our study, this character is only partially effective, and additional molecular analysis, morphological characters, and geographical distribution are necessary to delimit *Hyphodermella* species. In our ANOVA analysis (Fig. 5), *H. corrugata* and *H. salcedoae* have similar values for basidia and spore size; in this case, the macroscopy of the basidioma, the habitat, and the distribution can help differentiate them. The color change of the basidioma to wine red with KOH discriminates *H. ryvardenii* from the other species of the genus. This character is not uncommon in corticioid fungi and, in some cases, has been used to differentiate genera such as *Rhizochaete*, in which species turn red to purple in KOH (Greslebin et al. 2004), or to identify some species in *Candelabrochaete* (Dueñas et al. 2008) or *Xylodon* (Fernández-López et al. 2020).

By its morphological variability and wide distribution, *H. corrugata* has been considered a species complex (Telleria et al. 2010); our phylogenetic approach confirms that specimens reported from Argentina and Colombia (Hjortstam and Ryvarden 1986, 1997) and Australia (Hjortstam et al. 2009) correspond to two different species with restricted distributions. One specimen from Nepal, published as *H. corrugata* (Hjortstam and Ryvarden 1984), corresponds to an undescribed species of which we have studied only one specimen. These results suggest allopatric differentiation, supporting the importance of geographical isolation in fungal speciation, as in other organisms (Taylor et al. 2006).

*Hyphodermella* species are saprophytes that cause white rot in angiosperms, and little is known about their host specificity. It has been suggested that fungi with limited distributions are climate dependent or restricted to certain hosts (Ryvarden 1991). In this case, *H. maunakeaensis* and *H. salcedoae* have been reported growing on endemic species, *Myoporum acuminatum* and *Chamaecytisus proliferus*, from Hawaii and the Canary Islands, respectively. However, species with wide distributions, such as *H. corrugata* and *H. rosae*, have been reported on a wide variety of hosts.

Hjortstam and Ryvarden (1985) pointed out similarities between the mycobiota of southern South America and Australia and New Zealand, which has been confirmed in some corticioid fungi (Phookamsak et al. 2019; Fernández-López et al. 2019, 2020) and polyporoid fungi (Rajchenberg 2022). In this study, *H. corrugata* and *H. paulusiae* were reported from these areas, but, contrary to other cases, they are not phylogenetically related. According to Moncalvo and Buchanan (2008), when Southern Hemisphere species are identical to Northern Hemisphere species, human activity may be involved. The presence and abundance of *H. corrugata* in the Valdivian temperate forest of Chile may be explained by human translocation. Examples of fungi introduced by human activity are not rare, such as *Amanita muscaria*, a species native to boreal and temperate forests of the Northern Hemisphere that was introduced with exotic tree plantations in South America and is now widespread in native forests (Vargas et al. 2019), or *Favolaschia calocera*, introduced to New Zealand in the 1950s and now widely established, growing on native and exotic species (Johnston et al. 2006). Nevertheless, the presence of *H. corrugata* in southern South America could also be interpreted as an antitropical distribution pattern. Rajchenberg (2022) estimated that 17% of corticioid species in Patagonian forests of Argentina and 10% of polyporoid fungi in Andean forests of Chile and Argentina show this pattern.

## Conclusion

The morphological and multigene phylogenetic analyses support *Hyphodermella* as a monophyletic genus, with well-defined morphological characters: basidiome odontioid to hydnoid, hyphae without clamps, and encrusted cystidioid hyphae forming fascicles that sometimes project in the hymenium. We recognize in *Hyphodermella* the already described species *H. corrugata*, *H. maunakeaensis*, and *H. rosae*, as well as *H. paulusiae* from Australia and New Zealand, *H. ryvardenii* from Colombia and northern Argentina, and *H. salcedoae* from the Canary Islands, all described in this paper. We chose not to describe the singleton from Nepal until additional specimens become available to provide more information about its morphology.

## Supplementary Material

XML Treatment for Hyphodermella corrugata

XML Treatment for Hyphodermella maunakeaensis

XML Treatment for Hyphodermella paulusiae

XML Treatment for Hyphodermella rosae

XML Treatment for Hyphodermella ryvardenii

XML Treatment for Hyphodermella salcedoae

## Additional information

### Use of AI

No use of AI was reported.

### Author ORCIDs

Margarita Dueñas https://orcid.org/0000-0003-0621-8003

María P. Martín https://orcid.org/0000-0002-1235-4418

Sandra Nogal-Prata https://orcid.org/0000-0002-9430-5520

M.Teresa Telleria https://orcid.org/0000-0002-9876-6914

### Data availability

All of the data that support the findings of this study are available in the main text.
